# Innovative protocol of an exploratory study evaluating the acceptability of a humanoid robot at home of deaf children with cochlear implants

**DOI:** 10.1371/journal.pone.0285927

**Published:** 2023-06-16

**Authors:** Sabrina Stiti, Loïc Caroux, Pascal Gaillard, Pierre-Vincent Paubel, Olivier Deguine

**Affiliations:** 1 Laboratoire Cognition, Langues, Langage, Ergonomie (CLLE), Université Toulouse Jean Jaurès & CNRS, Toulouse, France; 2 Le Centre de Recherche Cerveau et Cognition (CerCo), Université Paul Sabatier & CNRS, Toulouse, France; 3 Service D’oto-rhino-laryngologie, Oto-neurologie et O.R.L Pédiatrique, CHU Toulouse, Hôpital Pierre-Paul Riquet, Toulouse, France; PLOS ONE, UNITED KINGDOM

## Abstract

The purpose of this paper is to introduce a research methodology for the assessment of the acceptability of a humanoid robot at home for children with cochlear implants (CI). The quality of audiology rehabilitation for cochlear implanted child administrated at the hospital with pluri-weekly sessions is a major prognostic factor in the outcome on communications abilities, but represents also a constraint for families related to the access to care that are more difficult. Further, home training with tools would balance the equitable distribution of care in the territory and promote the child’s progress. The humanoid robot should allow an ecological approach to this complementary training. Before developing this approach, it is necessary to study the acceptability of the humanoid robot at home, both by cochlear implanted child and their families. Ten families were chosen to have a humanoid robot at home, to explore their acceptability of the humanoid robot Pepper. The study lasts for 1 month per participants (i.e. cochlear implemented children and parent). Participants were invited to use the robot at home as much as they want. The humanoid robot Pepper was able to communicate and proposed activities not related to rehabilitation. Once a week during the study, data were collected from participants (questionnaires and robot’s logs) and the smooth running of the study was checked. Questionnaires are used to evaluate the acceptability of the robot by children and parents. User data from the robot’s logs are used to quantify the time and the actual use of the robot over the period of the study. Results of the experimentation will be reported, once all 10 participants have completed their passation. The robot is anticipated to be used and accepted by children with cochlear implants and their families.

**Clinical trial registration: ** Clinical Trials ID: NCT04832373; https://clinicaltrials.gov/.

## 1. Introduction

### 1.1. Rationale and background

According to the World Health Organization’s (WHO) world report on hearing, nearly 2.5 billion people worldwide will be living with some degree of hearing loss by 2050. Even in countries with relatively high proportions of ear and hearing care professionals, there is unequal distribution of specialists [1]. A medically underserved area is defined as a lack of access to practitioners, and the unequal distribution. Based on the WHO, around 34 million children worldwide live with disabling hearing loss, which affects their health and quality of life [1]. In this context, children with hearing impairment are one of the populations that suffer from medical desertification [1]. Cochlear implantation is the reference treatment for restoring hearing and promoting language development in children with total bilateral deafness that cannot be resolved. The invention of the cochlear implant has allowed deaf people to hear. It requires a surgical procedure, the indication for which is established through multidisciplinary consultation. More precisely, the cochlear implant is an instrument developed for people suffering from profound deafness. It provides access to sound using electrical stimulation to the auditory system. A child with a cochlear implant needs to learn how to identify sounds, locate their sources, recognize correlations between objects, and develop language abilities. The rehabilitation process for children with CI typically begins with learning to listen using the implant, without relying on lip-reading or with cued speech and then progressing to speaking and writing [2]. While speech therapy rehabilitation after surgery is effective, many parents struggle to continue this work at home. Family involvement in the rehabilitation process is a major prognostic factor for speech comprehension and language development after cochlear implantation [3–7]. However, children with cochlear implants may experience delays in the acquisition of reading, writing and social development, as well as deficits in visual-spatial-perceptual skills [6, 8]. Furthermore they tend to have low ability for problem-solving and logical thinking [3, 6, 8–11], which can impede their learning progress [7]. As a result, children with CI may require more attention and motivation for learning as they may become easily distracted and demotivated, when facing tasks that are beyond their ability level or that fail to capture their interest [9, 10, 12]. These difficulties have repercussions at the academic and social interaction [7, 13]. That’s the reason why, children with cochlear implants needs particular attention and a strict follow-up with evaluations and rehabilitations of hearing ability and speech intelligibility. Follow-up are carried out cooperatively by cochlear implantation team at hospital and speech therapist with pluri-weekly sessions in order for them to develop their cognitive abilities (e.g. auditory perception, speech comprehension and production, communication and language). Follow-up with pluri-weekly sessions represent a real constraint for families in term of time, number of rides and availabilities of families, so complementary home training work as a telemedicine service for speech comprehension and oral expression would balance the equitable distribution of care in the territory, decreased workload of families and should promote the child’s progress.

Even, the World Health Organization’s report on telemedicine, enhance that telemedicine has contributed to the quality and accessibility of health care [14]. Telemedicine can be defined by providing a medical care remote diagnosis and treatment of patients by means of telecommunications technology. For example, telemedicine has multiple applications and can be used for a variety of services, including wireless tools, email, two-way video and other methods of telecommunications technology. Telemedicine applications have successfully improved the quality and accessibility of medical care by allowing distant providers to evaluate, diagnose, treat, and provide follow-up care to patients [14, 15]. In fact, by increasing the accessibility of medical care, telemedicine can enable patients to seek treatment earlier and adhere better to their prescribed treatments [16], and improve the quality of life for patients with chronic conditions [14, 16–18] such as children with cochlear implants [19–22]. Indeed, cochlear implemented patients need to have their regular rehabilitation sessions, especially in infants and young children. For example, during the coronavirus (COVID-19) pandemic many parents couldn’t get an access to medical care for their children. Parents reported that cochlear implemented children rehabilitation was negatively affected [22]. In fact, the negative effect was mainly observed in their communication skills and ability of engagement in interactive activities [22]. If families could access to different types of telemedicine during pandemic or for areas lacking accessibility to medical care that would enhance follow-up of implanted children and avoid negative effect on their progress during rehabilitation.

So, one of a solution found to solve the lack of accessibility of medical care for implanted children, was the use of robots as one type of telemedicine that will provide a solution to the lack of practitioner and will solve mobility limitations [17, 23]. Indeed, social robots ’often represent technological solutions’, in the use of a technological approach to solve a pressing societal problem [24]. In the case of medical desertification, social robots will undeniably contribute to improving people’s quality of life [25]. In the field of health, robots are usually used to assist the independent living of people in situations of specific demand for assistance, to monitor the mental and physical well-being of patients [26, 27]. Social robots served as companionship, to provide comfort during check-up, increase well-being by reducing anxiety depression, fear, and pain, and also increase motivation, improve engagement, self-management and positive affect linked to healthcare intervention [23, 28–33].

So how social robots in healthcare as a telemedicine’s tool, would be fully accepted and trusted by people implicated in the healthcare system (patient, their family and care staff). That’s the reason why, the aim of this paper is to introduced a research methodology for the assessment of the acceptability of a social humanoid robot at home for children with cochlear implants and their family for a long stay of 1 month. The duration of one month was chosen based on Wu et al.’s [34] method, which allowed participants to become familiar with the robot and provide a more accurate evaluation of its performance.

First of all, considering every children (with and without impairments), the attribution of human-like characteristics to robots could enhance children’s understandings of usability and facilitated the children-robot interactions [35]. But a limit to the level of human-likeness should not be crossed otherwise might fall into Mori’s "uncanny valley" [36–38]. Mori’s theory postulates that the human appearance of robots is appreciated up to a certain point of similarity. Once the threshold is exceeded, to the point that the robot is so human-like in appearance as to be confusing, all the non-human aspects of the entity, triggered a feeling of strangeness. Thus, a robot with a human appearance will not be judged as a robot but as a human whose imperfections (slow movements, slow speech, wrinkles on the face) show that it is not acting in a normal way [38, 39]. The acceptability of social robots in various populations demonstrates that several types of factors need to be considered in order to foster user opinion and attitude [36, 37, 40, 41]. A review of the literature on the human response to assistive robots used in healthcare, shows that it is essential to consider both user factors and technology factors to promote acceptance [42]. There are 8 factors influencing user response to consider: age, needs, gender, experience with technology, cognitive skills, education level, anxiety level and initial attitude towards robots. In particular, participants’ initial attitude towards assistive robots would be a primary variable, with a participant’s positive attitude before an interaction with the technology being strongly correlated with a positive evaluation after the interaction [43].

Lastly, several researchers have investigated the use of robots for children who were deaf and implanted with cochlear implants or hearing aids [28, 44–47]. For example, according to Uluer et al. [48], the view of a robot in an interactive social environment, induce for users an expectation of a socially intelligent and socially conscious level of behavior based on the capabilities of the robot and the parameters of human-robot interactions [6]. However, most users are inexperienced with robots, and may have special needs like deaf children with CI. As Ioannou and Andreva showed the integration of a humanoid robot for hearing-impaired children, as a supportive tool for learning through play, have encouraging preliminary results [45]. Also robot movements are more effective than telling a story [45]. Children would also found the quality of the interaction with physically embodied robots more enjoyable than virtually embodied agents [44]. Indeed, having physical contact with the robot plays an important role in interactions with the child [49]. Also, humanoid robot affords numerous research opportunities and can be personalized for each child. In the case of the personalization of humanoid robots for children with cochlear implant, it is important to consider those elements of the robot that meet their needs. A balance is required between meeting these needs and the ability of the robot. It will enhance exchange, cooperation and engagement between the child and the robot. It also helps in building a relationship between two entities [50].

Therefore, robotic technologies are expected to fit certain social norms in order to facilitate interaction. Measurement of the acceptability of a humanoid robot for children with hearing disabilities, must be evaluated based on basic skills they have to interact, like Cano et al. [10] evaluation method for children with CI on interactive products. Which were direct observation of the child, thinking aloud, drawing intervention [51], identifying an image on a picture card, wizard of Oz, fun toolkit [52] and surveys. This indicates that these metrics may vary depending on the user profile (e.g. level of attention span and cognitive skills) and the purpose of the evaluation (assessing the user experience, the satisfaction or the usability). Basically, for deaf children with CI starting to develop their abilities to speak intelligibility, the thinking aloud method wouldn’t be a wise choice due to the need of verbalization while completing a task [53]. The picture card method would also be avoided for children with low ability of logical thinking but would be recommended for children with difficulties to speak because it doesn’t implicated verbalization [9, 10, 54]. The need to established evaluation methods adapted to the level of difficulty for deaf children with CI is crucial to assessed the child-robot interaction [9, 10, 12]. However, we were unable to find any studies that had investigated long-term interaction between deaf children with CI and a robot. Even studies about how to evaluate the level of acceptability of deaf children with cochlear implant. That’s the reason why, the aim of this paper is to introduced a methodology for the assessment of the acceptability of a social humanoid robot at home for children with cochlear implants and their family for a long period of time of 1 month.

### 1.2. Aim of the study

While development of telemedicine can help reduce medical desert and contribute to faster care and better quality for patients, the objective of this paper is to introduce a new framework of research methodology to evaluate the acceptability of a humanoid robot at home for deaf children with CI and their family members [55]. In this study, we firstly used questionnaires assessing the acceptability of the social robot weekly (usability, usefulness, playfulness, intention to use, attitude toward using the robot, etc.), for children with CI which were adapted for their level of language development and also for family members. And finally, user data from the robot were also recorded in real-time and weekly collected (user identification, date and hour of use, applications launched). This study aimed to investigate the use and acceptance of a humanoid robot during a one-month stay at home by deaf children with cochlear implants and their family members.

## 2. Materials and methods

### 2.1. Populations

Participants will be limited to children with cochlear implant and their parents or legal guardians. The selected children are part of the cohort followed regularly by the doctors and speech therapists of the pediatric cochlear implant unit (UPIC) of the Toulouse University Hospital. 10 families will be selected for this study. Recruitment of families started in September 2021 and is ongoing.

#### 2.1.1. Inclusion criteria

Children and their parents or legal guardians will be chosen amongst firstly, children who showed optimal use of the cochlear implant, and secondly, family with a favorable environment evaluated by speech therapists with Moeller’s scale for family involvement [56]. The following inclusion criteria for children will be used: (1) children aged between 8 to 12 years old. (2) The child must have at least one cochlear implant. (3) Surrounded by a supportive family environment, (4) undergo speech therapy and (5) be monitored by the pediatric cochlear implant unit (UPIC) of the Toulouse University Hospital.

#### 2.1.2. Exclusion criteria

The following exclusion criteria will be used: (1) children with cognitive or psychological incapacity; (2) refusal to give informed consent; (3) sensory or motor deficits that may interfere with the use of the robot; (4) an unstable psychiatric condition; (5) and a child whose two parents benefit from a measure of legal protection.

### 2.2. Ethics statement

Participants gave their written informed consent to participate in this study. Parents signed a confidentiality agreement and a non-disclosure agreement for the study. Their participation will be voluntary and will not affect the healthcare process. The entire study will be conducted in accordance with the Declaration of Helsinki and all relevant guidelines and regulations covering respect for the rights and dignity of participants. To protect confidentiality, all subjects will be given unique subject IDs which will be used for all study documentation. Authors will not get access to information that could identify individual participants during or after data collection. This research has been registered in ClinicalTrials.gov (https://clinicaltrials.gov/), April 5, 2021, under no. NCT04832373. The study is promoted by the University Hospital Center (CHU) of Toulouse. The Ethics Committee for Human Research in Ile de France Region 1 N°IRB: IORG0009918 (Paris, France) approved the study under number: 21.03768.041844-MS02. Written informed consent will be obtained for every participant.

### 2.3. Material

#### 2.3.1. Specifications of a humanoid Robot: Pepper

Considering the various factors that needed to be taken into account for the acceptability of social robots in different populations, the robot Pepper was chosen as an adequate option for the aim of study [33, 36, 37]. Pepper is an autonomous humanoid robot designed and created by the French company Aldebaran (acquired in 2015 by SoftBank Robotics) [57]. The version of Pepper used was 2.5.5 and is controlled by a Linux-based operating system called NAOqi. Physically, Pepper have a height of 1210mm and a weight of 27,82kg which makes Pepper small in size. Pepper was also selected because of a tablet of 10” fixed on its chest, which give a visual support and an alternative option to users, to answer in case of a lack of understanding by the robot. Pepper has 2 speakers, 4 microphones, ultrasonic sensors (2 transmitter and 2 receivers) and 2 cameras up to 640pp of resolution which is used by the robot for searching with his gaze and whole-body contacts with humans around. The *Autonomous Life mode* was enabled in order to give a more human like and natural behavior to the robot. The robot reacts to sounds and voices. The robot was able to speak, hear, and had facial and voice identification [58]. The robot was kept plug on its charger to disabled its wheels, so it wasn’t being able to drive, spin or turn. The lower body of the robot was immobilized in this study to avoid accidents and low battery alerts. The robot’s parameters were customized using Aldebaran’s Choregraphe 2.5.10.7 software which is used for programming and controlling of the robot.

#### 2.3.2. Personalization of Pepper

To enhance the interaction between the robot and each child with CI, the robot was personalized for each participant. It was designed to ask questions based on the child’s declared centers of interest during the inclusion process. The questions were implemented on the robot according to the child’s fields of interest and there were five themes with 10 questions per category that could only be activated by vocal command. An example is illustrated in Table 1.

**Table 1:** Example of customizable question themes implemented in Pepper, originally in French.

EXAMPLE OF THÈMES	QUESTIONS	PRE-REGISTRER ANSWER OF PEPPER
Climbing	How long have you been climbing?	Wow, I’m impressed!
	Do you train on boulders?	I’d love to see you climb one day
	What is the name of the first knot the climber ties?	That’s very interesting
	Do you attend any competitions?	I would love to be able to be present one day
Bike and soccer	How long have you been riding your bike?	Wow, I’m impressed.
	Do you practice playing soccer?	I’d love to see you play sometime
	Where do you like to go when you ride your bike?	I’d like to go for a ride together.
	Do you play soccer at school with your friends?	I would love to see that!
	What have you learned to do lately?	Wow, I’m impressed
	IN SOCCER, DO YOU PREFER TO PLAY IN ATTACK OR IN DEFENSE?	I WOULD BE A GOOD GOALKEEPER!

The robot was also personalized according to the rhythm of the family. Indeed, the robot was programmed with the family’s sleeping and meal times, which allowed it to adapt its activities and interactions accordingly. This personalization meant that the robot does not offer any activity during these times, so as not to disturb the rhythm of family life. He also makes remarks related to these times like "I’m getting hungry" at mealtimes or "I’m tired" at bedtime. The child also had the opportunity to ask general questions to the robot, to question the temporality (date and time of day) or the origins and function of the robot as well as to ask to make imitations of animals or sports for example. The robot can also tell some jokes and asks if the child is having fun. The speech rate was set to 80 words per minute (wpm) and the sounds settings were adjusted at home with the child. The child can also modify the "sound" setting to his liking with a command on the tablet or by asking the robot to increase or decrease the volume [59].

#### 2.3.3. Games and activities of Pepper on demand

In Table 2 below are described games and activities proposed by the robot and developed for participants.

**Table 2:** Description of games and activities of Pepper.

Name	Goal	Interaction mode
Hangman game	Participants had to find a word by guessing letters of the word. If that letter is in the word then write the letter in everywhere it would appear. If the letter wasn’t in the word then it added a body part to the gallows (head, body, left arm, right arm, left leg, right leg). Participants had to continue guessing letters until he can either solved the word or all six body parts were on the gallows	Vocal and/or tactile
Memory game	Participants had to find matching pairs of pictures	Tactile
Guess the animal	The robot gave hints to the child about an animal to find. If participants weren’t able to find, the robot will give a new hint. There were three chances to guess the animal otherwise the robot will give the answer	vocal and/or tactile
Complete the story	Invention of a story orally, based on a picture showed on the tablet, which was recorded by the robot	Vocal
Simple dances	The robot danced a choreography on songs that the child liked and listen often. At the end the robot asked the child if he wanted to continue by replaying the song or to ended the activity	Vocal and/or tactile
Imitation dances	The robot asked the child to imitated the choreography and it stopped during the song, and gave encouragement to the child to continue to dance along	Vocal and/or tactile

The robot recorded activity data in a log folder on its internal hard drive. The file made it possible to know the time and date of use, to identify the user and the start and end of an activity (games/dance/conversation).

### 2.4. Procedure

#### 2.4.1 Temporality of a passation

**Fig 1:**
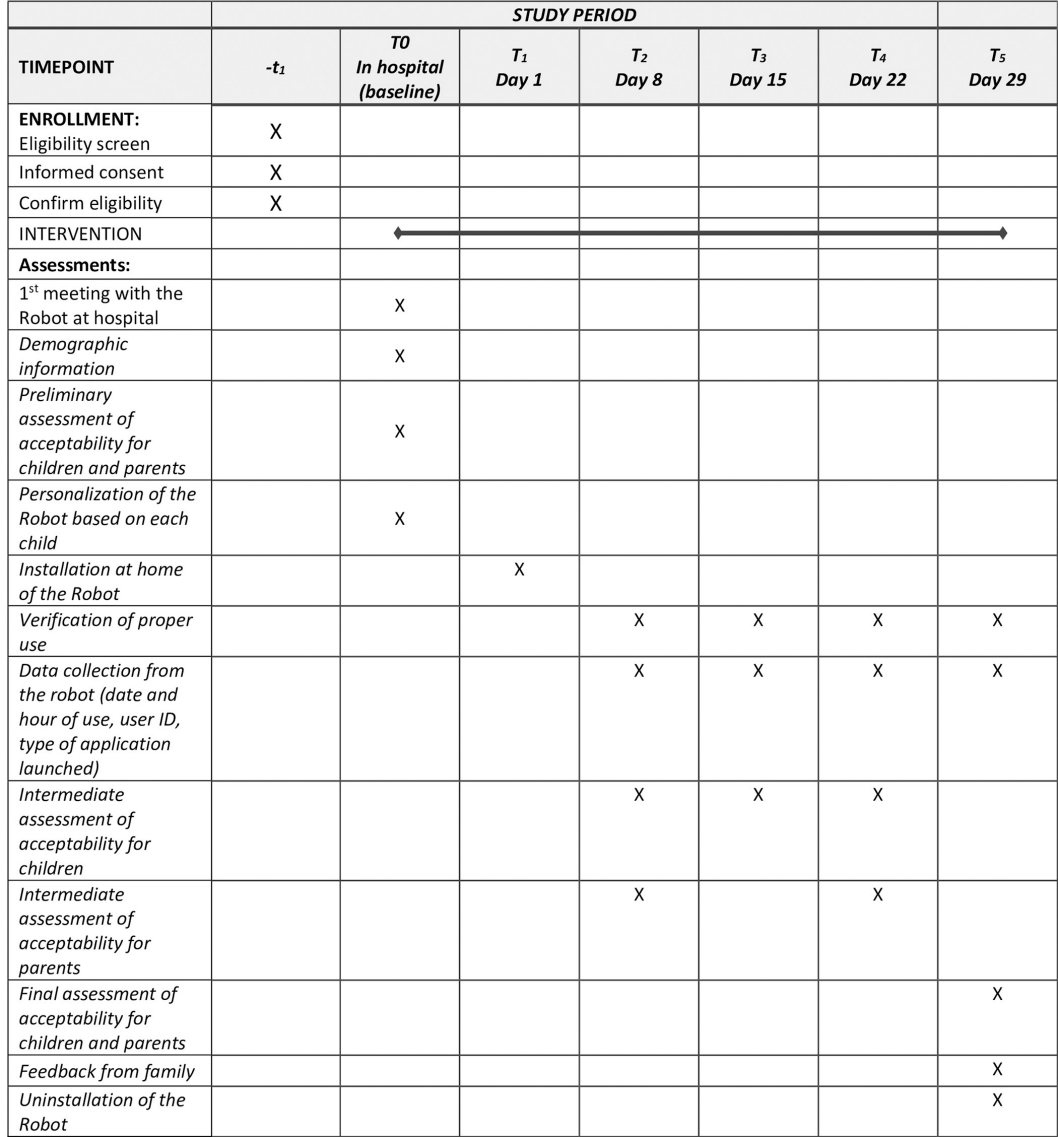
SPIRIT flow diagram: Schedule of enrolment, interventions, and assessment procedures.

**Fig 2:**
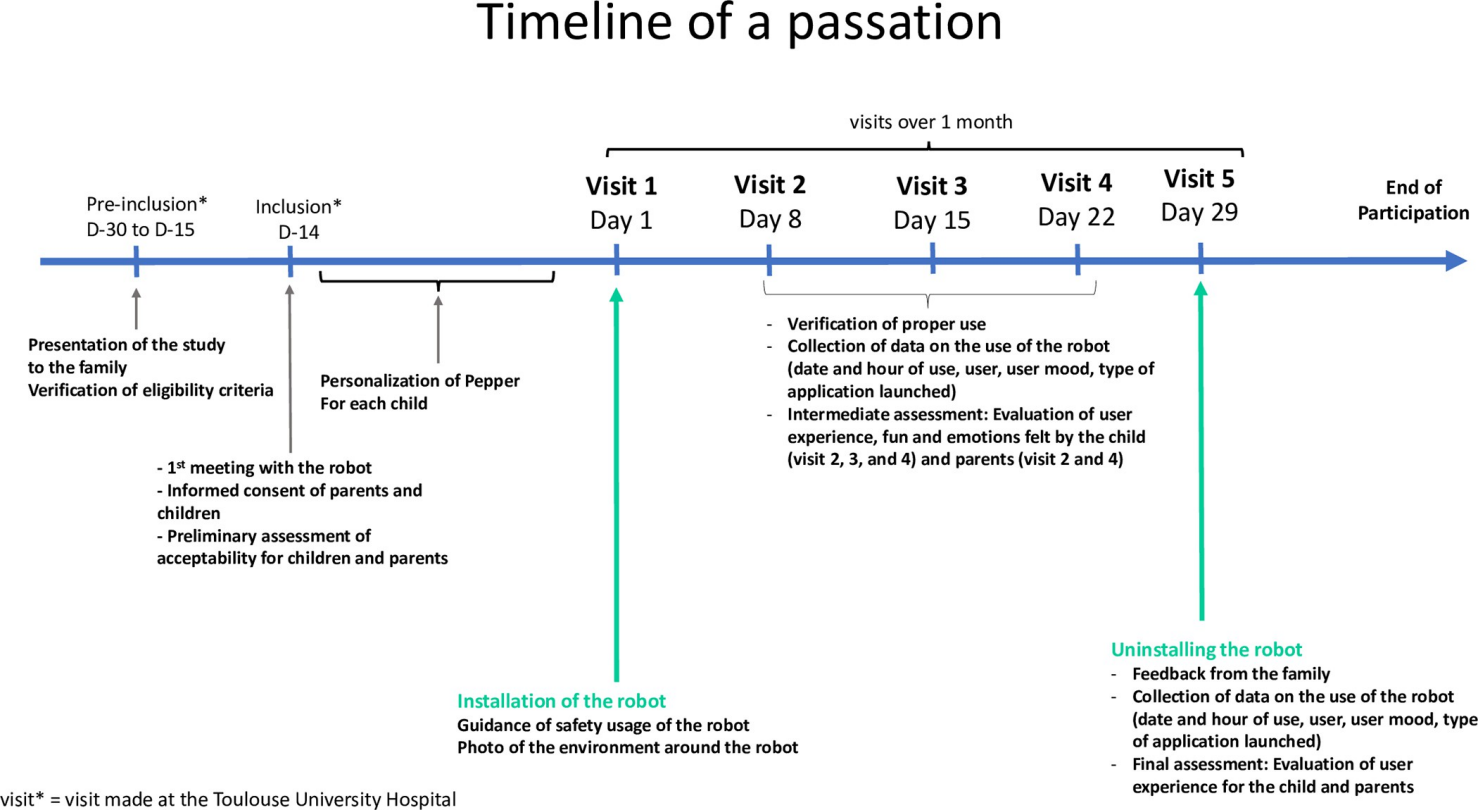
Timeline of a passation.

The Standard Protocol Items: Recommendations for Interventional Trials (SPIRIT) flow diagram schedule of enrolment, interventions and assessment procedures is provided in Fig 1. The temporality of the study takes place in several stages (Fig 2). First of all, the pre-inclusion, where the speech therapist from the ENT department in the pediatric cochlear implantation unit (UPIC) introduced the study to the family, checked the eligibility of the participants and explained the purpose of the study. Followed by the inclusion, where each parent and children signed a consent form before starting the study and then complete questionnaires of the preliminary assessment. Visit 1 was the installation of the robot at the participants home, for a period of one month (about 30 days). During this period, 4 visits (each week: visit 2, visit 3, visit 4 and visit 5) to the family’s home will be made to check if the study is running smoothly, to take data from the robot on a USB key and to retrieve questionnaires completed by the family, as well as the user’s oral feedback and the impression of the family. Visit 5 was marking the last day of a passation for participants with the uninstallation of the robot and the final assessment with questionnaires.

The chronology of the protocol creation began in 2018, and it took three years to select to population, develop questionnaires, select, and configure a humanoid robot suitable for deaf children with cochlear implants.

#### 2.4.2. Tasks during the stay of Pepper at home

The robot remained autonomously in participants home. The family and the child were instructed to use the robot at any time of the day and as many times as the child and the family wished. A society specialized in providing at home health care, was attributed to transport the robot at participants house and to visits them once a week, every week during the study (5 visits per participant). They will do three tasks: (1) check with the family if there is any problem or observation during the week with the robot at home; (2) collect data from the robot on a USB key thought the robot interface accessible on a computer with a RJ45 cable and the IP address of the robot; (3) give questionnaires to be completed by participants (children with cochlear implant and parents or legal guardians) during visits. It is important to note that questionnaires were administrated on paper and participants were given a sufficient amount of time to answer the questions (Table 3). Questionnaires were applied to children and parents as describe down below for visits and the details of questionnaires on the Table 3 and description on Table 5 for children and Table 6 for parents:

- **Inclusion:** Preliminary assessment for children and parents
- **Visit 2 to 4**: Intermediate assessment for children
- **Visit 2 and 4**: Intermediate assessment for parents
- **Visit 5:** Final assessment for parents and children

**Table 3:** Questionnaires applied for children and parents in preliminary intermediates and final assessment.

	1. Preliminary assessment (Inclusion)		2.Intermediaries assessment (Visit 2 to 4)		3.Final assessment (Visit 5)	
	Child	Parents	Child	Parents	Child	Parents
Drawing Intervention [51]	✓		✓		✓	
Use Intention Scale: Actual Use [60]				✓	✓	✓
Use Intention Scale: Attitude toward using [60]		✓		✓		✓
Use Intention Scale: Behavioral intentions to use [60]	✓	✓		✓	✓	✓
Adapted SUS [12]	✓		✓		✓	
meCue Module 1 [61]			✓	✓	✓	✓
meCue Module 2 [61]			✓	✓	✓	✓
meCue Module 3 [61]				✓		✓
meCue Module 4 [61]				✓		✓
Smiley-o-meter [62]			✓		✓	
The Again-Again table [62]			✓		✓	
Pick a mood robot [63]			✓		✓	
Pick a mood child [63]			✓		✓	
Heerink: the Almere Model [40]		✓		✓		✓
Personnal Innovativess [64]				✓		✓
AttrakDiff [65]		✓		✓		✓

#### 2.4.3. Measures

*2*.*4*.*3*.*1*. *Logs from the robot*. Data collection from the robot’s folder are time-stamped files which contain informational events relevant to the application. Therefore, logs contain transcription of activities done with the robot. The transcription of logs shows the name of the user, with dates and hours of use from the beginning till the end of the activity launched and the name of each activity initiated during the interaction. Logs are descripted in Table 4 with detail of which value were used to identify user interaction with the robot as a temporal analysis of the evolution of the use of the robot over the period of the study.

**Table 4:** Identification of logs retrieved from the robot and assessed measure.

Types of log analyzed	Attributes
Name of the user (name and tie in the family)	to identify users who had interactions with the robot (deaf children with CI or with participation of other family members (parents/siblings/friends/etc.)
Date of use per users (MM/DD/YYYY)	to identify actual days of use of the robot. Explore during the period of stay, days that appeared with more or less activities. To explore the evolution of use of the robot. To see if frequency of interaction with the robot increased or decreased per weeks.
Total length of use per activities per users (hh:mm:ss)	To identify which activities (with vocal and/or tactile command) were most used in time length per days and during the month. To explore the evolution of use of the robot. To see if it increased or decreased per days
Total length of use per days per users (hh:mm:ss)	To analyzed the increase or decrease of the total length of use per day on the robot’s one month stay.
Numbers of total launched per activities per users	To identify which activities (with vocal and/or tactile command) were most used and less used.

*2*.*4*.*3*.*2*. *Questionnaires for children with cochlear implant*. The purpose of using multiples questionnaires was to identify different aspects of the acceptability of the robot by assessing dimensions of usability, usefulness, playfulness, intention to use the robot, attitude toward using the robot, engagement of the participants (child with CI and parents) with the robot. This study was designed by dividing questionnaire of parents and children with CI. Considering the cognitive abilities of children with CI and the age range of 8 to 12 years old, we chose to use short questionnaires with sentences easy to understand (children with CI questionnaires were validated by speech therapists beforehand). Whereas parent questionnaire was chosen based on the evaluation of dimensions we aimed for (e.g. attractiveness to technology, innovative behaviors, intention to use, usability, acceptance). It was important to have data collected coming out of questionnaires from the child perception of the experience but also how parents perceived the robot. The child and their parents may have different perspectives on the interaction with the robot, and their feedback can complement and enrich each other. It would helped to have a more comprehensive understanding of the perception of the robot and the overall experience.

On Table 5 below, are described questionnaires proposed to children with cochlear implant to assessed the interaction between the child and the robot at home. Questionnaires were chosen in order to evaluated different aspect of perceived interaction to have detailed dimensions of the acceptability of the robot by children with CI and the evolution of results each week during the 1 month’s stay considering age, level of vocabulary and difficulties with verbal channel of communication. For example, drawing intervention [51] was selected as an alternative to verbalization and interview with the child with CI. Adding usability assessment and usefulness through Adapted System Usability Scale [12], Use Intention Scale [60] and meCue questionnaire [61] that were simplified with Likert-Smiley scale. Others dimensions such as mood (Pick-a-mood Child and Robot [63]), engagement (the Again-Again table [62]) and fun (Funometre [66, 67]) are assessed through visual channel of communication (as described in Table 5).

**Table 5:** Description of questionnaires for children with cochlear implant.

Questionnaires	Description
Drawing Intervention [51]	Assess without using orally or written words, the understanding of the robot. Children can communicate their experience with the robot at home through drawing
Use Intention Scale: Actual Use [60]	Measure the actual times and hours of use of the robot on a week on a 7-point Likert scale on a visual timeline with the end points being for the time of use “Not at all” and “Several times each day” and for hours “<1h” and “>25h”
Use Intention Scale: Behavioral intentions to use [60]	Measure the strength of willingness to use the robot a 7-point Likert-Smiley scale with the end points being “Strongly disagree” and “Strongly agree”. Version adapted to youth children by simplifying the syntax and vocabulary
Adapted System Usability Scale (SUS) [12]	Used to measure usability of the robot. This version was adapted for children between 4 to 6 years and for robot usage
meCue Module 1 [61]	Measure usefulness and usability on a 7-point Likert-Smiley scale with the end points being “Strongly disagree” and “Strongly agree”. Version adapted to youth children by simplifying the syntax and vocabulary
meCue Module 2 [61]	Measure positive emotions and negative emotions on a 7-point Likert scale with the end points being “Strongly disagree” and “Strongly agree”. Version adapted to youth children by simplifying the syntax and vocabulary
Funometre [66, 67]	A toolkit which measure the level of fun with children from “no fun at all” to “happy face” like a thermometer
The Again-Again table [62]	Measure the sustainability of an activity and the engagement felt during it. Activities are listed with the question “Would you like to do it again?” Possible answers were “Yes/Maybe/No”
Pick a mood robot [63]	Mood assessment toward the robot perceived by the child. Pictures of the robot represent different mood (i.e. Joyful/happy/apathetic/anxious/angry)
Pick a mood child [63]	Mood assessment of the child while interacting with the robot. Pictures children (boy/girl) represent different mood (i.e. Joyful/happy/ angry/scared/sad/tired)

*2*.*4*.*3*.*3*. *Questionnaires for parents*. Below are described questionnaires proposed to parents to assessed the interaction with the robot at home (Table 6). Questionnaires for parents were chosen in order to evaluated different aspect of perceived interaction to have detailed dimensions of the acceptability of the robot by children with CI and the evolution of results each week during the 1 month’s stay. Innovative behaviors [64] and attractiveness to technology [65] are measured to get a general understanding of parent’s technological profile. Ease of use, Usefulness and Playfulness through Use Intention Scale [60] are fundamental in determining the acceptance of the robot by measuring factor of actual length of use, Attitude toward using the robot and Behavioral intentions to use the robot [60]. The Almere Model [40] measure the acceptance of assistive social agent by following 13 constructs such as Attitude, Anxiety, Facilitating Conditions, Intention to Use, Perceived Adaptability, Perceived Enjoyment, Perceived Ease of Use, Perceived Sociability, Perceived Usefulness, Social Influence, Social Presence, Trust and Use. Finally, a modular evaluation of the parent User Experience [61] were used with 4 modules: Perception of Instrumental Qualities, User Emotions, Consequences of Use and a Global assessment of the robot.

**Table 6:** Description of questionnaires for parents.

Questionnaires	Description
Use Intention Scale: Actual Use [60]	Measure the actual times, hours and frequency of use of the robot on a 7-point Likert scale with the end points being for the time of use “Not at all” and “Several times each day”; for hours “<1h” and “>25h” and for the frequency of use “Extremely Infrequent” and “Extremely Frequent”.
Use Intention Scale: Attitude toward using [60]	Measure the attitude toward using the robot on a 7-point Likert scale with the end points being “good/bad””, “wise/foolish”, “pleasant/unpleasant” and “positive/negative”.
Use Intention Scale: Behavioral intentions to use [60]	Measure the strength of willingness to use the robot on a 7-point Likert scale with the end points being “Strongly disagree” and “Strongly agree”.
meCue Module 1: Perception of instrumental qualities [61]	Measure usefulness and usability on a 7-point Likert scale with the end points being “Strongly disagree” and “Strongly agree”.
meCue Module 2: User emotions [61]	Measure positive emotions and negative emotions on a 7-point Likert scale with the end points being “Strongly disagree” and “Strongly agree”.
meCue Module 3: Consequences of use [61]	Measure Intention to use and product loyalty a 7-point Likert scale with the end points being “Strongly disagree” and “Strongly agree”.
meCue Module 4: Global review [61]	Global review on a scale with the end points being -5 (bad) and 5 (good)
Heerink: the Almere Model [40]	Measure the acceptance of assistive social agent technology by older adults on a 5-point Likert scale with the end points being “Strongly disagree” and “Strongly agree”.
Personnal Innovativess [64]	Measure innovative behaviors in the domain of information technology on a 7-point Likert scale with the end points being “Strongly disagree” and “Strongly agree”.
AttrakDiff [65]	Measure the attractiveness to technology in the format of semantic differentials. Each set of adjective items is ordered into a scale of intensity on a 7-points Likert scale.

## 3. Results

The results of the experimentation are expected to be fully analyzed once all participants have completed their passation. Concerning the main criterion, the number of weekly hours of use of the robot will be described week by week for each patient, which will make it possible to describe the temporal evolution of the acceptability. The quantitative distribution of this indicator will be described in detail (number, mean, standard deviation, minimum, quartiles, median and maximum). A linear mixed model will be applied to estimate the average temporal evolution of the number of weekly hours of robot use in the whole study population. The same analysis approach will be applied form the results of the subjective assessment from questionnaires measured repeatedly during the follow-up: usability, acceptability, fun and emotions.

## 4. Perspectives

This article presents an original methodology that provides support for following the necessary guidelines to evaluate acceptability of a humanoid robot, adapted to the characteristics of children with cochlear implant and their family at home.

Our project proposed to develop an interactive, personalized solution of home training to help cochlear implanted children to progress in language expression and comprehension as a complement to regular speech therapy. This first step will provide essential data to improve knowledge in the field of the acceptability of a humanoid robot at home. In the long term, the development of a humanoid robot adapted to the training of deaf and implanted children at home will represent a therapeutic and technological breakthrough, since such a device could improve the quality of life and autonomy of these patients, and promote training, particularly in isolated areas.

## Supporting information

S1 File SPIRIT checklist. (DOC)

S2 File Questionnaires in original French version and the English translation: Preliminary assessment, intermediate assessment and final assessment for parents and children. (RAR)

S3 File Study Protocol approved by the Ethics committee in French and English. (PDF)
