# Supplementary material for: Innovative protocol of an exploratory study evaluating the acceptability of a humanoid robot at home of deaf children with cochlear implants
Source: PLoS One. 2023 Jun 16;18(6):e0285927. doi: 10.1371/journal.pone.0285927 (PMC10275444; doi:10.1371/journal.pone.0285927)
Supplement: S3 File — (PDF) [file pone.0285927.s003.pdf]

Evaluation de l’acceptabilité d’un robot humanoïde à domicile pour l'évaluation d'enfants implantés cochléaires

H2R2

PROTOCOLE DE RECHERCHE INTERVETIONNELLE IMPLIQUANT LA PERSONNE HUMAINE *catégorie 2 à risques et contraintes minimes*

Version n°*3.0 du 04/07/2022*

Code promoteur : RC31/20/0250

Numéro ID-RCB: 2020-A01989-30.

Cette recherche interventionnelle a obtenu le financement de la région Occitanie – Appel à projet « Recherche & Société 2018 »

Promoteur :
CHU TOULOUSE – Hôtel Dieu – 2, rue Viguerie – TSA 80035
31059 Toulouse cedex 9

Investigateur principal (recherche monocentrique) :
Pr DEGUINE Olivier
PU-PH
CHU Toulouse – Pierre Paul Riquet
Service d’ORL, ORL pédiatrique et Otoneurologie
Place du Dr Baylac
31059 Toulouse

Ce protocole a été conçu et rédigé à partir de la version 5.0 du 30/10/2017 du protocole-type du GIRCI SOHO

HISTORIQUE DES MISES A JOUR DU PROTOCOLE

VERSION	DATE	RAISON DE LA MISE A JOUR
1	29/01/2020	Demande de promotion au CHU de TOULOUSE
1.1	02/07/2020	Soumission initiale au CPP Sud-Med II
1.2	23/11/2020	Soumission initiale au CPP IDF 1
1.3	05/02/2021	Réponse aux commentaires du CPP IDF 1
2.0	28/10/2021	Modification substantielle n°1
3.0	04/07/2022	Modification substantielle n°2

PAGE DE SIGNATURE DU PROTOCOLE

Evaluation de l'acceptabilité d'un robot humanoïde placé à domicile chez un enfant implanté cochléaire

H2R2

Code promoteur : RC31/20-0250

**Promoteur**<br><br>CHU de TOULOUSE<br>Hôtel Dieu<br>2, rue Viguerie TSA 80035<br>31059 TOULOUSE cedex 9<br>Tel : +33 (0)5 61 77 86 03<br>Fax : +33 (0)5 61 77 84 11<br>*drci.toulouse@chu-toulouse.fr*	Fait à Toulouse,<br><br>Le	Mr Olivier LAIREZ,<br>Directeur de la Recherche et de l'Innovation<br>*signature*
**Investigateur principal**<br><br>*Pr DEGUINE Olivier*<br>*PU-PH*<br>*CHU Toulouse – Pierre Paul Riquet*<br>*Service d'ORL, ORL pédiatrique et Otoneurologie*<br>*Place du Dr Baylac*<br>*31059 Toulouse*<br>Courriel : *deguine.o@chu-toulouse.fr*	Fait à Toulouse<br><br>Le	Pr DEGUINE Olivier<br>PU-PH<br><br>*signature*

PRINCIPAUX CORRESPONDANTS

Investigateur principal
Pr DEGUINE Olivier
CHU Toulouse – Pierre Paul Riquet
Service d'ORL, ORL pédiatrique et Otoneurologie
Place du Dr Baylac
31059 Toulouse

Investigateur associé
Dr Calmels Marie Noelle
Service ORL, Otoneurologie et ORL pédiatrique
Hôpital Pierre Paul Riquet
Place du Dr Baylac
31059 Toulouse
Tel : 05 61 77 90 09

Dr Baladi Blandine
Service ORL, Otoneurologie et ORL pédiatrique
Hôpital Pierre Paul Riquet
Place du Dr Baylac
31059 Toulouse

Pr Marx Mathieu
Service ORL, Otoneurologie et ORL pédiatrique
Hôpital Pierre Paul Riquet
Place du Dr Baylac
31059 Toulouse

Dr Yohan Gallois
Service ORL, Otoneurologie et ORL pédiatrique
Hôpital Pierre Paul Riquet
Place du Dr Baylac
31059 Toulouse

Autres spécialités
Cochard Nadine (orthophoniste)
Husson Hélène (orthophoniste)
Lasfargues Anne (orthophoniste)
Hôpital Pierre Paul Riquet
Place du Dr Baylac
31059 Toulouse

Unité de vigilance de la recherche clinique
Dr Pascale OLIVIER-ABBAL
Service de pharmacologie médicale et clinique & Direction de la Recherche, du Développement et de l'Innovation
CHU TOULOUSE
Tel : 05 61 14 59 98 (CRPV)
Tel : 05 61 77 85 56 (DRDI)

Maison des Sciences de l'Homme et de la société
Pierre Vincent Paubel
Université Toulouse Jean Jaurès
5 Allée Antonio Machado
31058 Toulouse cedex 9

Laboratoire Cognition, Langues, Langage, Ergonomie- UMR 5263 - CNRS
Pascal Gaillard
Loïc Caroux
Maison de la Recherche
Université Toulouse Jean Jaurès
5 Allée Antonio Machado
31058 Toulouse cedex 9

Centre de Recherche Cerveau et Cognition – UMR 5549
Pascal Barone
Kuzma Streilnikov
Chloe Farrer
Sabrina STITI
Pavillon Baudot
Purpan
31059 Toulouse

Accompagnement projet
Institut des Technologies Avancées et chirurgicales (ITAC)
Chef de projets : Aline MEULLE
CHU Toulouse – Hôpital Pierre Paul Riquet
Hall C – 2ème étage
Place du Dr Baylac
31059 Toulouse

Promoteur
CHU TOULOUSE – Hôtel
Dieu – 2, rue Viguerie – TSA
80035
31059 Toulouse cedex 9

Responsable recherche : Dr
Marie-Elise LLAU
Tel : 05 61 77 86 03

ARC réglementaire : Florine LEGAY

Centre de Méthodologie
Unité de Soutien à la Recherche Clinique
(USMR)
Service de Pharmacologie - Faculté de
Médecine - 37 allées Jules Guesde
31000 Toulouse

Méthodologiste : Benoit Lepage

SOMMAIRE

Page de signature du protocole 3
SOMMAIRE 6
1. RESUME DE LA RECHERCHE 9
2. JUSTIFICATION SCIENTIFIQUE ET DESCRIPTION GENERALE 12
2.1. ETAT ACTUEL DES CONNAISSANCES 12
2.1.1. Sur la pathologie 12
2.1.2. Sur les traitements/stratégies/procédures de référence et à l'étude 12
2.2. HYPOTHESES DE LA RECHERCHE ET RESULTATS ATTENDUS 12
2.3. JUSTIFICATION DES CHOIX METHODOLOGIQUES 12
2.4. RAPPORT BENEFICE / RISQUE 12
2.5. RETOMBEES ATTENDUES 13
2.6. JUSTIFICATION DU FAIBLE NIVEAU D'INTERVENTION 13
3. OBJECTIFS DE LA RECHERCHE 14
3.1. OBJECTIF PRINCIPAL 14
3.2. OBJECTIFS SECONDAIRES 14
4. CRITERES DE JUGEMENT 14
4.1. CRITERE DE JUGEMENT PRINCIPAL 14
4.2. CRITERES DE JUGEMENT SECONDAIRES 14
CONCEPTION DE LA RECHERCHE 16
4.3. SCHEMA DE LA RECHERCHE 16
5. CRITERES D'ÉLIGIBILITE 16
5.1. CRITERES D'INCLUSION 16
CRITERES DE NON INCLUSION 16
5.2. FAISABILITE ET MODALITES DE RECRUTEMENT 16
6. TRAITEMENT(S)/STRATEGIE(S)/PROCEDURE(S) DE LA RECHERCHE 17
6.1. TRAITEMENT/STRATEGIE/PROCEDURE EXPERIMENTAL(E) 17
7. TRAITEMENTS ET PROCEDURES ASSOCIE(E)S 17
7.1. TRAITEMENTS/PROCEDURES ASSOCIE(E)S AUTORISE(E)S 17
7.2. TRAITEMENTS/PROCEDURES ASSOCIE(E)S INTERDIT(E)S 17
8. DEROULEMENT DE LA RECHERCHE 18
8.1. CALENDRIER DE LA RECHERCHE 18
8.2. TABLEAU RECAPITULATIF DU SUIVI PARTICIPANT 18
8.3. VISITE DE PRE INCLUSION & D'INCLUSION 19
8.3.1. Recueil du consentement 19
8.3.2. Déroulement de la visite d'inclusion 19
8.4. VISITES A DOMICILE DE SUIVI J21, J28, J36 19
8.5. VISITE DE FIN DE LA RECHERCHE 20
8.6. REGLES D'ARRET DE LA PARTICIPATIONS D'UNE PERSONNE A LA RECHERCHE 20
8.7. CONTRAINTES LIEES A LA RECHERCHE ET INDEMNISATION EVENTUELLE DES PARTICIPANTS 20
9. GESTION DES ÉVÉNEMENTS INDÉSIRABLES / EFFETS INDESIRABLES / INCIDENTS 20

10. ASPECTS STATISTIQUES 21
10.1. CALCUL DE LA TAILLE D'ETUDE 21
10.2. METHODES STATISTIQUES EMPLOYEES 21
11. SURVEILLANCE DE LA RECHERCHE 22
12. DROITS D'ACCES AUX DONNEES ET DOCUMENTS SOURCE 22
12.1. ACCES AUX DONNEES 22
12.2. DONNEES SOURCE 22
12.3. CONFIDENTIALITE DES DONNEES 22
13. CONTROLE ET ASSURANCE QUALITE 23
13.1. CONSIGNES POUR LE RECUEIL DES DONNEES 23
13.2. CONTROLE QUALITE 23
13.3. GESTION DES DONNEES 23
13.4. AUDIT ET INSPECTION 24
14. CONSIDERATIONS ETHIQUES ET REGLEMENTAIRES 24
15. CONSERVATION DES DOCUMENTS ET DES DONNEES RELATIFS A LA RECHERCHE 25
16. RAPPORT FINAL 25
17. REGLES RELATIVES A LA PUBLICATION 25
17.1. COMMUNICATIONS SCIENTIFIQUES 25
17.2. COMMUNICATION DES RESULTATS AUX PARTICIPANTS 26
17.3. CESSION DES DONNEES 26
REFERENCES BIBLIOGRAPHIQUES 27

LISTE DES ABREVIATIONS

ANSM	Agence Nationale de Sécurité du Médicament et des produits de santé
CPP	Comité de Protection des Personnes
EvI	Evènement Indésirable
EvIG	Evènement Indésirable Grave
EIG	Effet Indésirable Grave
EIGI	Effet Indésirable Grave Inattendu
SUSAR	Suspected Unexpected Serious Adverse Reaction

1. RESUME DE LA RECHERCHE

**PROMOTEUR**	CHU Toulouse
**INVESTIGATEUR COORDONNATEUR/PRINCIPAL**	Pr DEGUINE Olivier<br>CHU Toulouse – Pierre Paul Riquet<br>Service d'ORL, ORL pédiatrique et Otoneurologie<br>Place du Dr Baylac<br>31059 Toulouse
**TITRE**	Evaluation de l'acceptabilité d'un robot humanoïde placé à domicile chez un enfant implanté cochléaire –H2R2
**JUSTIFICATION / CONTEXTE**	La qualité de la réhabilitation de la surdité chez l'enfant implanté cochléaire est un facteur pronostique majeur du résultat sur la compréhension de la parole et l'expression orale. Cette réhabilitation est effectuée conjointement par l'équipe hospitalière d'implantation cochléaire, et par un/une orthophoniste situé(e) à proximité du domicile de l'enfant. Les séances pluri hebdomadaires représentent une contrainte pour l'enfant et ses parents liée à la disponibilité indispensable, aux déplacements, et à l'anxiété générée par le milieu non écologique en cabinet ou en hôpital. De plus, certains territoires sont insuffisamment pourvus en orthophonistes, rendant l'accès aux soins plus difficile. Un travail complémentaire d'entraînement à domicile permettrait d'équilibrer l'équité de répartition de soins dans le territoire, et devrait favoriser les progrès de l'enfant, plus enclin à utiliser un outil disponible à domicile. Le robot humanoïde devrait permettre une approche écologique de ce complément de réhabilitation. Avant de développer cette démarche, il est nécessaire d'étudier l'acceptabilité du robot humanoïde à domicile, à la fois par les parents et par l'enfant implanté cochléaire.
**OBJECTIFS**	L'objectif principal est d'évaluer l'acceptabilité par l'enfant et sa famille, d'un robot humanoïde installé à domicile pendant un mois.<br><br>Les objectifs secondaires sont<br>- décrire l'acceptabilité de l'enfant et de sa famille vis-à-vis du rapport à la technologie, de l'intention d'usage, des attentes, de l'utilité perçue, de la perception du robot et des conditions facilitatrices avant puis après 1 mois d'utilisation du robot à domicile,<br>- décrire l'acceptabilité de l'enfant vis-à-vis de son expérience, de son amusement et de ces émotions ressenties lorsqu'il utilise le robot à domicile
**CRITERES DE JUGEMENT**	**<u>Critères de jugement principal</u>**<br>L'acceptabilité d'un robot humanoïde à domicile, par l'enfant et par sa famille sera évaluée par le nombre d'heures hebdomadaire durant lesquelles l'enfant sollicite le robot, à domicile.<br><br>**<u>Critères de jugement secondaires</u>**<br>Les critères de jugement des objectifs secondaires sont les suivants<br><br>• Les réponses aux questionnaires et échelles standardisés<br>• La complétion de phrase<br>• Le « pick-a-mood » version émotion. La catégorisation et le classement des activités entre-elles<br>• L'entretien semi-directif

**SCHEMA DE LA RECHERCHE**	Etude monocentrique prospective en ouvert d'évaluation de l'acceptabilité d'un robot humanoïde PEPPER à domicile d'enfants sourds avec implant cochléaire.
**CRITERES D'INCLUSION**	- Enfant âgé de 8 à 12 ans, utilisateur de son implant cochléaire avec un entourage familial favorable,<br>-Enfant implanté avec au moins un implant cochléaire en cours de réhabilitation orthophonique, et suivis par l'unité pédiatrique d'implants cochléaires (UPIC) du CHU de Toulouse<br>- Enfant et sa famille dont le français est la langue maternelle<br>-Affiliation à un régime de la sécurité sociale
**CRITERES DE NON INCLUSION**	- Inaptitude cognitive ou psychologique ou refus du participant de donner son consentement écrit<br>- Autre déficit sensoriel ou moteur pouvant interférer dans l'utilisation du robot<br>- Pathologie psychiatrique instable<br>- Enfant dont les deux parents bénéficient d'une mesure de protection juridique
**TRAITEMENTS/STRATEGIES/PROCEDURES DE LA RECHERCHE**	Pepper est un robot humanoïde mesurant 1,20 m de haut. Il s'adapte parfaitement à l'utilisation par des enfants car il a la taille d'un humain de 8 ans.<br><br>Pour l'étude, nous disposons de deux robots PEPPER qui auront été préalablement programmés et implémentés avec les tests de l'étude. Les robots seront mis à disposition des patients de façon alternée.<br>A ce jour, les 2 robots ont été implémentés avec les éléments suivants :<br>• Reconnaissance des personnes : identification faciale<br>• Personnalisation pour l'enfant<br>• Personnalisation au rythme de la famille<br>• Activités<br>• Interaction<br><br>Aucune donnée en dehors de celles nécessaires pour répondre aux objectifs de l'étude ne sera enregistrée ou sauvegardée. De plus, le robot n'a pas de connexion à distance et n'est pas relié à internet. Aucune donnée ne peut être télétransmise. Les données seront récupérées par clef USB lors des visites de suivi.<br><br>Une fois le robot mis à la disposition de l'enfant à son domicile, il pourra le solliciter autant de fois qu'il le souhaite et à n'importe quel moment de la journée
**TAILLE D'ETUDE**	N=10
**DUREE DE LA RECHERCHE**	Durée de la période d'inclusion : 18 mois<br>Durée effective de participation de chaque participant : 1 mois<br>Durée totale de participation de chaque participant : environ 1 an et 1 mois<br>Durée totale de la recherche (durée de la période d'inclusion + durée de participation) : 20 mois
**ANALYSE STATISTIQUE DES DONNEES**	Une analyse descriptive des critères de jugement sera réalisée. Un modèle linéaire mixte sera appliqué pour estimer l'évolution temporelle moyenne du nombre d'heures hebdomadaires de sollicitation du robot
**RETOMBEES ATTENDUES**	Notre projet propose de mettre en place une solution interactive, personnalisée pour aider les enfants implantés cochléaires à progresser dans l'expression et la compréhension du langage en complément de la prise en charge orthophonique habituelle. Cette

	première étape fournira des données essentielles permettant d'améliorer les connaissances dans le domaine de l'acceptabilité à domicile d'un robot humanoïde. A terme, le développement à domicile d'un robot humanoïde adapté à la rééducation d'enfants sourds et implantés représentera une avancée thérapeutique et technologique, puisqu'un tel dispositif pourrait améliorer la qualité de vie et l'autonomie de ces patients, et favoriser la réhabilitation, en particulier dans les territoires isolés.

2. JUSTIFICATION SCIENTIFIQUE ET DESCRIPTION GENERALE

2.1. ETAT ACTUEL DES CONNAISSANCES

2.1.1. SUR LA PATHOLOGIE

L'implantation cochléaire est le traitement de référence pour restaurer l'audition et le développement du langage chez l'enfant présentant une surdité totale bilatérale non appareillable. Elle nécessite un acte chirurgical, dont l'indication est posée en concertation multidisciplinaire. La réhabilitation orthophonique post opératoire, et l'implication de la famille dans cette réhabilitation sont des facteurs pronostiques majeurs pour la compréhension de la parole et le développement du langage après implantation cochléaire.

2.1.2. SUR LES TRAITEMENTS/STRATEGIES/PROCEDURES DE REFERENCE ET A L'ETUDE

La réhabilitation après implantation cochléaire est organisée de manière coordonnée entre l'équipe hospitalière d'implantation cochléaire pédiatrique (UPIC), et le relai assuré par une orthophoniste située à proximité du domicile de l'enfant. Elle suppose des séances pluri-hebdomadaires d'entrainement auditif et d'expression orale. Les visites régulières au centre d'implantation sont nécessaires pour vérifier l'état et la bonne utilisation de l'implant, contrôler les réglages et réaliser un bilan orthophonique. Ce bilan permet d'apprécier les progrès de l'enfant en terme de compréhension et d'expression de la parole, d'adapter et de guider la réhabilitation aux capacités de l'enfant.

Cette réhabilitation orthophonique peut être limitée par les contraintes qu'elle impose aux familles. Ces contraintes sont liées à la nécessité d'une disponibilité importante pour accompagner l'enfant chez l'orthophoniste ou à l'Hôpital, aux transports itératifs. Dans certains cas l'éloignement géographique dans les territoires sous médicalisés peut amplifier ces contraintes. De plus, la fatigue, les contraintes horaires, le stress lié à l'environnement médical ou paramédical peuvent avoir un effet négatif chez l'enfant implanté.

L'utilisation d'un robot humanoïde de type NAO a été utilisé avec succès chez des enfants présentant des troubles autistiques comme le décrit Diehl et al et Ismail et al.

Le robot Pepper a été peu utilisé dans les structures de soins. Il a montré son utilité pour l'accueil de personnes dans des structures commerciales ou industrielles. Son aspect rassurant, sa reconnaissance faciale et sa morphologie contribuent à le rendre acceptable par des personnes non initiées à l'approche robotique.

2.2. HYPOTHESES DE LA RECHERCHE ET RESULTATS ATTENDUS

Nous postulons que le robot humanoïde PEPPER utilisé à domicile sera bien accepté par l'enfant sourd et sa famille.

2.3. JUSTIFICATION DES CHOIX METHODOLOGIQUES

Schéma de la recherche : A ce stade très précoce de l'évaluation de l'utilisation du robot Pepper à domicile, nous proposons une étude pilote sur un petit nombre de patients, suffisant pour répondre à des critères d'évaluation de l'acceptabilité.

Choix de l'objectif principal : L'acceptabilité d'un robot humanoïde à domicile est un préalable indispensable à la mise en œuvre d'une aide à la réhabilitation, chez l'enfant sourd implanté cochléaire.

Choix de la population : Les enfants sélectionnés font partie de la cohorte suivie régulièrement par les médecins et orthophonistes de l'UPIC. Ils seront choisis parmi ceux qui présentent une utilisation optimale de l'implant cochléaire, avec un entourage familial favorable.

2.4. RAPPORT BENEFICE / RISQUE

BENEFICES INDIVIDUELS

La sollicitation à domicile du robot par l'enfant, à tout moment de la journée et autant de fois que l'enfant le désire, réduit les contraintes liées à la réhabilitation en cabinet ou à l'hôpital. La proposition de jeux "éducatifs" orientés sur le langage permet de libérer l'enfant du stress des séances orthophoniques, en respectant son rythme de vie. Il s'agit d'une réhabilitation personnalisée en milieu naturel, ce qui est

difficilement réalisable avec les méthodes habituelles ; on peut espérer que l'enfant en retirera un bénéfice sur ses capacités de compréhension et d'expression orale.
Il s'agit d'un exemple typique de médecine personnalisée et participative, au service du patient et de sa famille.

BENEFICES COLLECTIFS
Cette étude apportera des données primordiales sur l'acceptabilité d'utiliser à domicile des robots humanoïdes chez des enfants implantés cochléaires. Démontrer l'acceptabilité de l'utilisation de la robotique à domicile permettra une avancée majeure pour faciliter le parcours de soins et réduire les inégalités territoriales dans les zones sous médicalisées.
Les résultats de cette étude d'acceptabilité pourront aboutir en cas de résultats positifs, à la mise en place d'études évaluant l'efficacité de l'utilisation d'un robot humanoïde dans la réhabilitation du patient implanté cochléaire et plus généralement du patient en situation de handicap. Cette information sera particulièrement utile pour les personnes à mobilité réduite.

RISQUES
Il s'agit d'un robot marqué CE commercialisé, utilisé couramment pour les interactions Homme/robot. Son utilisation rentre dans le cadre de sa conception. Il n'y a pas de risque connu d'utilisation de cet outil.

CONTRAINTES
Les contraintes liées à la recherche sont considérées comme minimes. La participation à l'étude nécessitera 4 visites à domicile d'environ 1h – 1h30 par l'investigateur ou son représentant ; la contrainte est donc liée à la disponibilité de la famille pendant cette visite. L'enfant sera libre d'utiliser le robot quand il le souhaite, et pour la durée qu'il souhaite.
De plus, l'enfant et ses parents devront compléter divers questionnaires lors des visites. Le temps de complétion de ces questionnaires varie entre 20 et 75 minutes.

Le rapport bénéfice / risque peut donc être jugé comme très favorable.

2.5. RETOMBEES ATTENDUES

Notre projet propose de mettre en place une solution interactive, personnalisée pour aider les enfants implantés cochléaires à progresser dans le développement du langage compris et parlé, en complément de la prise en charge orthophonique habituelle. Cette première étape fournira des données essentielles permettant d'améliorer les connaissances dans le domaine de l'acceptabilité à domicile d'un robot humanoïde. A terme, le développement à domicile d'un robot humanoïde adapté à la rééducation d'enfants sourds et implantés représentera une évolution thérapeutique et technologique, en rendant un dispositif de réhabilitation disponible en milieu écologique (le domicile), qui pourra être sollicité sans limite, au rythme choisi par l'enfant et sa famille.

2.6. JUSTIFICATION DU FAIBLE NIVEAU D'INTERVENTION

On peut considérer qu'il s'agit d'un protocole de recherche générant des risques et contraintes minimes pour le patient (catégorie 2 RIPH), conformément à l'arrêté du 12 avril 2018 fixant la liste des recherches à risques et contraintes minimes :
«2. Administration ou utilisation de produits mis sur le marché au sein de l'Union européenne, lorsque les conditions d'utilisation de ces produits sont conformes à leur destination et à leurs conditions d'utilisation courante.
11. Entretiens, observations et questionnaires dont les résultats, conformément au protocole, peuvent conduire à la modification de la prise en charge médicale habituelle du participant et ne relevant pas de ce fait de la recherche mentionnée au 3° de l'article L. 1121-1 du code de la santé publique. »

La contrainte de temps en lien avec le protocole de recherche correspond à 1h - 1h30 d'entretien à domicile, toutes les semaines pendant un mois, ainsi que le temps de complétion des questionnaires (de 20 à 75 minutes selon la visite)

Les risques en lien avec le protocole de recherche peuvent être considérés comme minimes, et correspondent aux risques d'utilisation du robot. Le robot, marqué CE, est utilisé pour réaliser des tests d'orthophonie et des jeux à l'enfant. Les conditions d'utilisation du robot dans le projet sont conformes à son utilisation courante puisque le robot Pepper est conçu pour interagir avec l'homme.

3. OBJECTIFS DE LA RECHERCHE

3.1. OBJECTIF PRINCIPAL

L'objectif principal est d'évaluer l'acceptabilité par l'enfant et par sa famille, d'un robot humanoïde placé pendant un mois à domicile.

3.2. OBJECTIFS SECONDAIRES

Les objectifs secondaires sont

- de décrire l'acceptabilité de l'enfant et de sa famille vis-à-vis du rapport à la technologie, de l'intention d'usage, des attentes, de l'utilité perçue, de la perception du robot et des conditions facilitatrices avant puis après 1 mois d'utilisation du robot à domicile,
- de décrire l'acceptabilité de l'enfant vis-à-vis de son expérience, de son amusement et de ces émotions ressenties lorsqu'il utilise le robot à domicile

4. CRITERES DE JUGEMENT

4.1. CRITERE DE JUGEMENT PRINCIPAL

L'acceptabilité d'un robot humanoïde à domicile, par l'enfant et par sa famille sera évaluée par le nombre d'heures hebdomadaire durant lesquelles l'enfant sollicite le robot.
Chaque fois que le robot sera sollicité, le temps d'utilisation sera enregistré.

4.2. CRITERES DE JUGEMENT SECONDAIRES

Les critères de jugement des objectifs secondaires sont les suivants :

- Des questionnaires et échelles standardisés validés par la communauté scientifique évaluent le rapport à la technologie, l'intention d'usage, la relation avec le robot et l'expérience utilisateur. Ils permettent à la fois de comparer les réponses des différents participants entre eux, mais aussi celles d'un même participant à deux moments donnés. La complétion de phrase est utilisée pour recueillir les attentes des participants. Il s'agit d'une technique utilisée dans le domaine de l'UX (expérience utilisateur) qui consiste à donner un début de phrase à l'utilisateur et à le laisser compléter librement la suite. Cela permet d'avoir des données un peu plus subjectives et complémentaires à celles obtenues via les questionnaires. Les enfants passeront le SUS (Systeme Usability Scale) qui est un questionnaire UX standardisé et validé qui mesure l'utilisabilité du robot par l'enfant. La version utilisée a été adapté pour les enfants par Sanchez-Morales et al.,2020 [(15)]. Il comprend 10 phrases affirmatives. Une analyse des dessins faits par l'enfant va être réalisée à l'aide d'une grille de cotation dessin (méthode de dessins de XU ET AL, 2009)[16].
- Un « pick-a-mood » version émotion de l'enfant (fille/garçon), adaptée de la version « pick-a-mood » émotion perçue du robot, permettra d'évaluer les émotions de l'enfant.
- La catégorisation et le classement des activités entre-elles par les enfants : effectuée selon divers critères (bon fonctionnement, amusement, facilité, entre autres) elle permet d'avoir un retour sur le contenu implémenté sur le robot.
- L'entretien semi-directif : prévu comme dernier recueil de cette expérience il doit permettre d'une part d'obtenir un retour plus détaillé et libre de la part des parents, mais aussi de l'enfant implanté. Les parents et l'enfant participeront à un entretien distinct lors de la désinstallation du robot.

L'ensemble des questionnaires et méthodes utilisés est détaillé dans le tableau ci-dessous :

Questionnaires standardisés		
**Attrakdif [14]**	Mesure du rapport à la technologie en général	Parents
**Personal innovativness [1]**	Rapport à la technologie	Parents/Enfant
**Use Intention Scale [16]**	Intention d'usage	Parents/Enfant
	Utilisation effective	Enfant
**Heerink questionnaire [9]**	Utilité perçue, anxiété, attitude d'usage, conditions facilitatrices, intention d'usage, adaptivité perçue, appréciation perçue, facilité d'utilisation perçue, sociabilité perçue, influence sociale, présence sociale, confiance	Parents
**MeCUE [13]**	Expérience utilisateur, intention d'usage, utilité perçue,	Enfant
SUS [15]	utilisabilité, influence sociale, satisfaction	Enfant
**Echelles**		
**Fun-o-meter [17]**	Amusement	Enfant
**Pick-a-mood [7]**	Emotions perçue du robot	Enfant
**Autres méthodes**		
**Complétion de phrases [12]**	Attentes, attitude	Parents/Enfant
**Classement des activités [17]**	Amusement, satisfaction, préférences dans les activités	Enfant
**Catégorisation des activités [17]**	Intention d'usage spécifique pour les activités	Enfant
**Entretien**	Relation robot/enfant, recueil de subjectivité	Parents/Enfant
**Recueil d'informations**	Expérience passée, rapport à la technologie	Parents/Enfant

CONCEPTION DE LA RECHERCHE

4.3. SCHEMA DE LA RECHERCHE

Il s'agit d'une étude pilote monocentrique prospective en ouvert d'évaluation de l'acceptabilité d'un robot humanoïde PEPPER au domicile d'enfants porteurs d'un implant cochléaire.

5. CRITERES D'ÉLIGIBILITE

5.1. CRITERES D'INCLUSION

Les critères d'inclusion sont les suivants :
- Enfant âgé de 8 à 12 ans, utilisateur de son implant cochléaire avec un entourage familial favorable,
- Enfant implanté avec au moins un implant cochléaire en cours de réhabilitation orthophonique, et suivi par l'unité pédiatrique d'implants cochléaires (UPIC) du CHU de Toulouse
- Enfant et sa famille dont le français est la langue maternelle
- Affiliation à un régime de la sécurité sociale

CRITERES DE NON INCLUSION

Les critères d'exclusion sont les suivants :
- Inaptitude cognitive ou psychologique ou refus du participant de donner son consentement écrit
- Autre déficit sensoriel ou moteur pouvant interférer dans l'utilisation du robot
- Pathologie psychiatrique instable
- Enfant dont les deux parents bénéficient d'une mesure de protection juridique

5.2. FAISABILITE ET MODALITES DE RECRUTEMENT

Les enfants sont recrutés dans le service ORL, Otoneurologie et ORL pédiatrique du CHU de Toulouse lors d'une consultation dans le cadre de leur suivi orthophonique.

Ce service est spécialisé dans la prise en charge de la surdité pédiatrique et a une grande expérience de la recherche clinique. La file active est de 410 enfants implantés cochléaires, en progression constante (28 par an en 2015, 39 par an en 2019), ce qui est plus que suffisant pour assurer le recrutement des 10 patients pour notre étude de faisabilité.

Dans le cadre de ce protocole de recherche, seuls les patients déjà présents dans la file active des soins courants sont recrutés, ce qui facilite le recrutement et limite le nombre de perdus de vus. Le nombre de perdus de vus devrait également être limité en raison du déroulement du protocole qui met à disposition un robot au domicile du patient : cette mise à disposition implique une surveillance régulière de la bonne utilisation du robot par l'équipe projet. Dans ce cadre, Des photos non identifiantes de l'environnement d'installation du robot seront faites et la société installant le robot renseignera certains paramètres tels que la présence d'animaux de compagnie ou la disposition du robot.

En pratique, les investigateurs proposent aux enfants et à leur parents/représentants légaux présentant l'ensemble des critères d'inclusion, sans critère de non-inclusion, de participer au protocole de recherche H2R2.

Le parcours des patients correspond à celui de la prise en charge habituelle :

- La première visite est la visite d'inclusion et permet au patient de se familiariser avec le robot
- Le robot est installé au domicile de l'enfant pendant 1 mois selon la disponibilité des parents et du robot. L'enfant peut interagir avec autant de fois qu'il le souhaite. Durant ce mois, l'investigateur ou son représentant passera toutes les semaines au domicile du patient pour s'assurer de la bonne utilisation du robot et recueillir les difficultés rencontrées et le vécu par la famille et l'enfant.

La prise en charge pour la réhabilitation orthophonique reste inchangée pendant la période de participation à l'étude de l'enfant.

6. TRAITEMENT(S)/STRATEGIE(S)/PROCEDURE(S) DE LA RECHERCHE

6.1. TRAITEMENT/STRATEGIE/PROCEDURE EXPERIMENTAL(E)

Pepper est un robot humanoïde mesurant 1,20 m de haut. Il s'adapte parfaitement à l'utilisation par des enfants car il a la taille d'un humain de 8 ans. Il dispose de 17 articulations qui permettent un langage corporel, de 3 roues omnidirectionnelles pour se déplacer facilement. Il atteint au maximum la vitesse moyenne de marche d'un humain. Il dispose d'une tablette tactile, permettant une interaction manuelle complétant l'interaction vocale. Le robot parle, entend, et possède une identification faciale.
Il a une autonomie de plus de 12 heures d'énergie pour des activités en continu. La tablette est compatible avec un large type de contenus : vidéos, applications, textes, boutons, etc. Cet accessoire devient une véritable interface supplémentaire pour interagir avec le robot Pepper.

Pour l'étude, nous disposons de deux robots PEPPER qui auront été préalablement programmés pour répondre aux besoins de l'étude. Les robots seront mis à disposition des patients de façon alternée. Entre chaque patient, le robot restera un mois à la MSHS pour permettre à l'équipe de le préparer pour le patient suivant.

A ce jour, les 2 robots ont été implémentés avec les éléments suivants :

- <u>Reconnaissance des personnes</u> : Le robot enregistre le prénom et le visage des membres de la famille et les reconnait. Il s'adresse aux personnes en utilisant leur prénom et il peut enregistrer de nouveaux visages si on le lui demande. Le robot fait également la distinction entre l'enfant implanté et les autres et ne propose des activités qu'à l'enfant implanté ; néanmoins tout le monde peut interagir avec lui.
- <u>Personnalisation pour l'enfant</u> : Le robot peut poser quelques questions sur les centres d'intérêt déclarés par l'enfant en amont. Il peut aussi faire des remarques en lien avec ces sujets au cours de l'interaction.
- <u>Personnalisation au rythme de la famille</u> : le robot connaît les heures de coucher et de repas de l'enfant et ne propose pas d'activité durant ces moments, pour ne pas perturber le rythme familial. Il fait également des remarques liées à ces moment (ex : « je commence à avoir faim » à midi).
- <u>Activités</u> : Le robot propose 3 jeux (pendu, memory et devinettes d'animaux), une activité d'histoire à compléter, des danses et des danses à imiter.
- <u>Interaction</u> : Le robot peut répondre à des questions d'ordre général sur lui, la temporalité (jour, heure) et la surdité. Le robot peut aussi raconter quelques blagues. Le robot propose à l'enfant de faire une pause au bout d'un certain temps d'interaction et il lui demande aussi s'il s'amuse.

A noter que le robot n'utilise pas son micro pour enregistrer des données dans l'environnement familial. Aucune donnée en dehors de celle nécessaire pour répondre aux objectifs de l'étude ne seront enregistrée ou sauvegardée. De plus, le robot n'a pas de connexion à distance et n'est pas relié à l'internet. Aucune donnée ne peut ainsi être transmise. Le recueil des données utiles au projet (temps d'utilisation) seront récupérées par clef USB lors des visites de suivi.

Ces caractéristiques ont été implémentées dans le robot par les ingénieurs de la Maison des Sciences de l'Homme et de la Société ainsi que par les chercheurs du laboratoire CLLE.

Une fois le robot mis à la disposition de l'enfant à son domicile, il pourra le solliciter autant de fois que voulu et à n'importe quel moment de la journée.

7. TRAITEMENTS ET PROCEDURES ASSOCIE(E)S

7.1. TRAITEMENTS/PROCEDURES ASSOCIE(E)S AUTORISE(E)S

Les enfants pourront continuer à prendre leur traitement habituel.

7.2. TRAITEMENTS/PROCEDURES ASSOCIE(E)S INTERDIT(E)S

Aucun traitement ni de procédure sont interdits

8. DEROULEMENT DE LA RECHERCHE

8.1. CALENDRIER DE LA RECHERCHE

- Durée de la période d'inclusion : 18 mois
- Durée effective de participation de chaque participant : 1 mois
- Durée totale de participation de chaque participant : 1 ans et 1 mois
- Durée totale de la recherche : 20 mois

8.2. TABLEAU RECAPITULATIF DU SUIVI PARTICIPANT

	Pré *inclusion (0 à 15 jours avant V0)*	Inclusion[1] V0	Visite 1 *V1 : J1 (installation)*	Visite 2 *V2 : J8+2 Après installation*	Visite 3 *V3 : J15+2 Après installation*	Visite 4 *V4 : J22+2 Après installation*	Visite 5 V5 : J29+2 *Après installation*
	CONS.	CONS.	DOMICILE				
Notice d'information *(R)*	✓	✓					
Consentement éclairé *(R)*		✓					
Vérification des critères d'éligibilité *(R)*	✓	✓					
Installation du robot à domicile (R)			✓				
Désinstallation du robot (R)							✓
Evaluation préliminaire de l'acceptabilité (rapport à la technologie, intention d'usage, attentes, utilité perçue, perception du robot, condition facilitatrices) chez l'enfant et sa famille (R)		✓					
Evaluation intermédiaire : Evaluation de l'expérience utilisateur, l'amusement et les émotions ressenties par l'enfant (R)				✓	✓	✓	
Evaluation intermédiaire : Evaluation de l'expérience utilisateur, les émotions ressenties par les parents (R)				✓		✓	
Retour d'expérience final : Evaluation de l'expérience utilisateur chez l'enfant et sa famille (R)							✓
Collecte des données du robot sur une clé USB (date et heure d'utilisation, utilisateur, humeur de l'utilisateur, type d'application lancée)			✓	✓	✓	✓	✓
Utilisation du robot par l'enfant			✓ (autant de fois que l'enfant le voudra)				

[1] Délai maximum estimé entre la signature du consentement et l'installation du robot d' 1an +/- 2 mois.
CONS consultation

8.3. VISITE DE PRE INCLUSION & D'INCLUSION

Dans le cadre du suivi de l'enfant sourd profond (possédant un implant cochléaire fonctionnel), la famille est vu en consultation par un médecin investigateur dans le service d'oto-rhino-laryngologie (ORL) à l'hôpital Pierre Paul Riquet (PPR) au CHU de Purpan. Lors de cette consultation et après s'être assuré de l'éligibilité de l'enfant, le médecin investigateur informera la famille de l'étude et leur proposera d'y participer. La notice d'information de l'étude sera remise aux familles.

8.3.1. Recueil du consentement

Lors de la visite d'inclusion, un des médecins investigateurs informe l'enfant et les représentants de l'autorité parentale et répond à toutes leurs questions concernant l'objectif, la nature des contraintes, les risques prévisibles et les bénéfices attendus de la recherche. Il précise également les droits de l'enfant dans le cadre d'une recherche interventionnelle impliquant la personne humaine et vérifie les critères d'éligibilité.

Le médecin investigateur est responsable de l'obtention du consentement éclairé écrit des représentants de l'autorité parentale. Le formulaire de consentement doit être signé avant toute procédure prévue par le protocole de la recherche.

Si les représentants de l'autorité parentale donnent leurs accords de participation, ces derniers et l'investigateur inscrivent leurs noms et prénoms en clair, datent et signent le formulaire de consentement. Bien que seule l'autorisation des titulaires de l'autorité parentale soit requise, il ne sera pas passé outre le refus de participation du mineur.

Conformément à la Loi Jardé pour les recherches de catégorie 2, il sera possible de ne recueillir le consentement que d'un seul titulaire de l'autorité parentale pour les enfants sourds, si le consentement de l'autre titulaire ne peut être recueilli dans les délais impartis par le protocole de recherche. Les différents exemplaires de la note d'information et du formulaire de consentement sont alors répartis comme suit :
- Un exemplaire de la note d'information et du consentement signé est remis aux titulaires de l'autorité parentale
- L'exemplaire original est conservé par le médecin investigateur (même en cas de déménagement du patient pendant la durée de la recherche) dans un lieu sûr inaccessible à des tiers.

8.3.2. Deroulement de la visite d'inclusion

La visite d'inclusion est assurée par le médecin investigateur. Avant tout examen lié à la recherche, l'investigateur recueille le consentement libre, éclairé et écrit du participant (ou de son représentant légal le cas échéant).

Une première évaluation du rapport à la technologie, l'intention d'usage, les attentes, l'utilité perçue, la perception du robot ainsi que les conditions facilitatrices est réalisée avant l'interaction avec celui-ci. Ces éléments seront mesurés à la fois chez l'enfant implanté et chez ses parents. La complétion de ces questionnaires durera entre 20 et 30 minutes pour les parents et entre 40 et 50 minutes pour l'enfant.

Le robot Pepper sera ensuite présenté à l'enfant afin qu'il puisse se familiariser avec son utilisation. L'investigateur expliquera à l'enfant et sa famille le fonctionnement du robot et les différentes interactions que peux avoir l'enfant avec lui.

Le robot sera ensuite installé pendant 1 mois au domicile de l'enfant selon la disponibilité des parents et du robot . Une société de prestation sera en charge d'installer et de désinstaller le robot au domicile des patients. Le robot sera transporté dans sa mallette de transport et les prestataires devront s'assurer de son bon fonctionnement. Les prestataires pourront aussi répondre aux questions techniques des patients si besoin.

La société de prestation sera spécialisée dans le domaine de la santé et habilitée à réaliser ce type de prestation. Les prestataires n'auront pas accès aux données médicales des patients.

8.4. VISITES A DOMICILE DE SUIVI J8+2, J15+2, J22+2, J29+2

L'installation du robot au domicile de l'enfant est à J1.

Les visites de suivis auront lieu toutes les semaines au domicile de l'enfant par un investigateur ou son représentant (employés de la société de prestation, habilités à réaliser ce genre de missions). Lors de cette visite, l'expérience utilisateur, l'amusement et les émotions ressenties par l'enfant implanté en lien avec son utilisation du robot seront évalués. La complétion du questionnaire par l'enfant durera entre 20 et 25 minutes. L'expérience utilisateur, l'amusement et les émotions ressenties par le parent seront évalués à J8+2 et J22+2.

La complétion du questionnaire par le parent durera entre 20 et 25 minutes. La bonne utilisation du robot sera également vérifiée.

La mise en place du robot par la société de prestation et son enlèvement seront soumis aux règles d'hygiène en vigueur : port du masque, respect de la distanciation physique, désinfection du robot.
Des photos non identifiantes de l'environnement d'installation du robot seront faites

Le robot sera désinstallé au cours de la visite à J29+2.

8.5. VISITE DE FIN DE LA RECHERCHE

La visite de fin de la recherche correspond à la désinstallation du robot à J29+2 . Elle aura lieu au domicile de l'enfant avec un investigateur ou son représentant. Lors de cette visite, une évaluation finale du rapport à la technologie, l'intention d'usage, les attentes, l'utilité perçue, la perception du robot ainsi que les conditions facilitatrices est réalisée après 1 mois d'utilisation à domicile. Ces éléments seront mesurés à la fois chez l'enfant implanté et chez ses parents. Un entretien semi directif sera réalisés afin d'obtenir un retour plus détaillé et libre de la part des parents et de l'enfant. La complétion du questionnaire ainsi que l'entretien dureront entre 45 et 55 minutes pour les parents et entre 65 et 75 minutes pour l'enfant.

8.6. REGLES D'ARRET DE LA PARTICIPATIONS D'UNE PERSONNE A LA RECHERCHE

L'arrêt définitif de la participation d'une personne à la recherche pourra s'effectuer dans les cas suivants :

- Décision délibérée du patient la participation d'un patient à la recherche s'arrêtera en cas de retrait du consentement ;
- En raison du décès de la personne ;
- Le sujet est perdu de vue : si le patient n'est plus suivi dans l'étude sans qu'aucune cause ne soit repérée après une investigation active, il est alors perdu de vue.

Si un patient arrête prématurément la recherche, la fiche de fin d'essai sera complétée pour le patient et les raisons de sa sortie de l'étude consignées.

Si certains participants arrêtent la recherche avant la fin de l'étude, ils seront remplacés.

En cas d'arrêt prématuré de la recherche, d'exclusion de la recherche, de retrait du consentement ou d'abandon de la recherche, la prise en charge se poursuivra selon les modalités habituelles, dans le cadre de leur parcours de soins courants.

8.7. CONTRAINTES LIEES A LA RECHERCHE ET INDEMNISATION EVENTUELLE DES PARTICIPANTS

Les patients inclus ont interdiction de participer simultanément à une autre recherche interventionnelle dans le but d'éliminer toute interférence. Il n'y a pas de période d'exclusion à la fin de l'étude.

Aucune indemnisation ne sera versée au patient.

9. GESTION DES ÉVÉNEMENTS INDÉSIRABLES / EFFETS INDESIRABLES / INCIDENTS

Ce protocole correspond à une recherche impliquant la personne humaine « à risques et contraintes minimes » (catégorie 2 selon l'article L1121 du CSP) qui n'oblige plus les investigateurs à déclarer les événements indésirables « graves » au promoteur.
La procédure de l'étude (utilisation d'un robot humanoïde) est dénuée de risques.

Ainsi, les EIG ne seront pas à déclarer au promoteur.

L'investigateur doit signaler les éventuels effets indésirables ou incidents potentiels aux différents **réseaux de vigilance sanitaire** applicables à chaque produit ou procédure concernés

(pharmacovigilance, vigilance des dispositifs médicaux, hémovigilance, vigilance de soins standards…) conformément à la réglementation en vigueur.

En cas de notification d'un effet indésirable/ incident, l'investigateur doit préciser que le patient est inscrit à un essai clinique et identifier clairement l'essai clinique concerné.

En cas d'effet indésirable potentiel ou de survenue d'un incident pouvant affecter de manière significative la sécurité des patients et/ou la balance bénéfices/risques et/ou la conduite de l'étude H2R2, l'investigateur avertira immédiatement le promoteur aux coordonnées mentionnées ci-dessous :

.

Promoteur : CHU de TOULOUSE
Nom du responsable de la vigilance : Pascale OLIVIER-ABBAL
Fax : 05 61 77 84 11
Courriel : vigilance.essaiscliniques@chu-toulouse.fr

10. ASPECTS STATISTIQUES

10.1. CALCUL DE LA TAILLE D'ETUDE

Si on considère comme critère d'acceptabilité le fait « d'utiliser le robot pendant au moins 1h par semaine », on espère que ce critère d'acceptabilité puisse être atteint par au moins la moitié des participants. On considère également que si moins de 15% des patients atteignent ce temps moyen d'utilisation, l'acceptabilité est clairement insuffisante pour cette version du robot.

On peut appliquer un plan de Fleming A'Hern en une seule étape, où nous souhaitons pouvoir rejeter l'hypothèse nulle (A'Hern RP. Sample size tables for exact single-stage phase II designs. Stat med 2001;20:859-66):

H0 = {Pr(utiliser le robot pendant au moins 1h par semaine) ≤ 15%),

avec une hypothèse alternative possible :

H1 = {Pr(utiliser le robot pendant au moins 1h par semaine) ≥ 50%),

avec une puissance de 80% et un risque α de 5%.

Le nombre de patients nécessaires à inclure est de 10 patients, et on rejetterait l'hypothèse nulle si au moins 4 patients sur un total de 10 utilisent le robot pendant au moins 1h par semaine.

En d'autres termes, si dans un échantillon de 10 patients, moins de 4 patients utilisent le robot pendant au moins 1h par semaine, l'acceptabilité sera considérée comme insuffisante pour poursuivre l'évaluation du robot dans cette version vers des études comparatives de plus haut niveau de preuve.

10.2. METHODES STATISTIQUES EMPLOYEES

Les données manquantes seront décrites et les données aberrantes recherchées à l'aide de contrôles logiques. Ces données manquantes et aberrantes seront corrigées dans la mesure du possible. L'analyse se fera sur l'ensemble des participants ayant accepté de participer à l'étude.

Un flow-chart sera réalisé pour décrire les inclusions et les sorties prématurées éventuelles (avec les motifs de sortie).

Concernant le critère de jugement principal, le nombre d'heures hebdomadaires de sollicitation du robot sera décrit semaine après semaine pour chaque patient, ce qui permettra de décrire l'évolution temporelle de

l'acceptabilité. La distribution quantitative de cet indicateur sera décrite de manière détaillée (effectif, moyenne, écart type, minimum, quartiles, médiane et maximum).
Un modèle linéaire mixte sera appliqué pour estimer l'évolution temporelle moyenne du nombre d'heures hebdomadaires de sollicitation du robot dans l'ensemble de la population d'étude.

La même démarche d'analyse sera appliquée pour les critères de jugement mesurés de manière répétée au cours du suivi : expérience utilisateur, amusement et émotions ressenties.

Concernant les critères de jugement secondaires mesurés à la fin d'étude, une analyse descriptive simple de la distribution sera réalisée (effectif, moyenne, écart type, minimum, quartiles, médiane et maximum).

11. SURVEILLANCE DE LA RECHERCHE

Etant donné le caractère minime des risques et contraintes de l'étude, aucun comité de surveillance ne sera constitué Cependant un comité scientifique composé de l'équipe investigatrice, du promoteur et du méthodologiste pourra se réunir afin de suivre l'avancée des inclusions et discuter d'éventuelles difficultés rencontrées.

12. DROITS D'ACCES AUX DONNEES ET DOCUMENTS SOURCE

12.1. ACCES AUX DONNEES

L'acceptation de la participation au protocole implique que les investigateurs mettront à disposition les documents et données individuelles strictement nécessaires au suivi, au contrôle de qualité et à l'audit de la recherche, à la disposition des personnes ayant un accès à ces documents conformément aux dispositions législatives et réglementaires en vigueur.

12.2. DONNEES SOURCE

Ensemble des informations figurant dans des documents originaux, ou dans des copies authentifiées de ces documents, relatif aux examens cliniques, aux observations ou à d'autres activités menées dans le cadre d'une recherche et nécessaires à la reconstitution et à l'évaluation de la recherche. Les documents dans lesquels les données sources sont enregistrées sont appelés les documents sources.

Dans le cadre de cette recherche les documents sources seront le dossier médical des patients, les données recueillies sur le robot et sur tablette. Les résultats de l'évaluation orthophonique seront répertoriés dans le cahier d'observation de ce fait ces données n'auront pas de données de source.

12.3. CONFIDENTIALITE DES DONNEES

Conformément aux dispositions législatives en vigueur, les personnes ayant un accès direct aux données source prendront toutes les précautions nécessaires en vue d'assurer la confidentialité des informations relatives aux médicaments expérimentaux, aux recherches, aux personnes qui s'y prêtent et notamment en ce qui concerne leur identité ainsi qu'aux résultats obtenus. Ces personnes, au même titre que les investigateurs eux-mêmes, sont soumises au secret professionnel.

Pendant la recherche ou à son issue, les données recueillies sur les personnes qui s'y prêtent et transmises au promoteur par les investigateurs (ou tous autres intervenants spécialisés) seront rendues anonymes. Elles ne doivent en aucun cas faire apparaître en clair les noms des personnes concernées ni leur adresse.

Seules la première lettre du nom et du prénom du sujet seront enregistrées, accompagnées d'un numéro d'inclusion.

Le promoteur s'assurera que chaque personne qui se prête à la recherche a donné son accord par écrit pour l'accès aux données individuelles la concernant et strictement nécessaires au contrôle de qualité de la recherche.

13. CONTROLE ET ASSURANCE QUALITE

13.1. CONSIGNES POUR LE RECUEIL DES DONNEES

Toutes les informations requises par le protocole doivent être consignées sur les cahiers d'observation et une explication doit être apportée pour chaque donnée manquante. Les données doivent être recueillies au fur et à mesure qu'elles sont obtenues, et transcrites dans ces cahiers de façon nette et lisible.

Les données sont recueillies sur un cahier d'observation papier

13.2. CONTROLE QUALITE

Un attaché de recherche clinique mandaté par le promoteur visite de façon régulière chaque centre investigateur, lors de la mise en place de la recherche, une ou plusieurs fois en cours de recherche selon le rythme des inclusions et en fin de recherche. Lors de ces visites, et conformément au plan de monitorage basé sur le risque (participant, logistique, impact, ressources), les éléments suivants seront revus :

- consentement éclairé,
- respect du protocole de la recherche et des procédures qui y sont définies,
- qualité des données recueillies dans le cahier d'observation : exactitude, données manquantes, cohérence des données avec les documents source (dossiers médicaux, carnets de rendez-vous, originaux des résultats de laboratoire, etc,…),

Toute visite fera l'objet d'un rapport de monitorage par compte-rendu écrit.

13.3. GESTION DES DONNEES

L'investigateur s'assure que l'anonymat de chaque personne participant à l'étude est garanti. Aucune information permettant l'identification des personnes n'est communiquée à des tiers autres que ceux réglementairement habilités à détenir cette information (et qui sont tenus au secret professionnel).

Les informations sont recueillies pour chaque sujet sur un cahier d'observation rempli par les investigateurs du centre et les orthophonistes. Les données cliniques et comportementales recueillies seront reportées sur un cahier d'observation papier.

Un document source (dossier médical existant et cahier d'observation créé pour les besoins de l'étude) est tenu pour chaque sujet participant à l'étude ; l'observation et le suivi médical concernant l'étude seront consignés dans ce document. Ce document source sera conservé dans le service d'ORL de l'Hôpital Pierre Paul Riquet.

Toutes les données de l'étude sont conservées sur support informatique par les médecins investigateurs ou leurs délégués.

Le cahier d'observation de chaque patient sera conservé au service d'ORL de l'Hôpital Pierre Paul Riquet sous le contrôle du médecin investigateur principal, et fera l'objet d'une revue par l'ARC de monitoring sur site, avant d'être adressé au Technicien d'Etude pour saisie.

La saisie des données sera supervisée par le méthodologiste et le statisticien de l'étude. Les modalités de saisie seront définies conjointement avec le Responsable du projet.

Les données recueillies sur le robot seront conservées sur support informatique externe en respectant les méthodes d'anonymat de l'étude. Ces données seront conservées pour une durée de 15 ans minimum.

L'analyse statistique sera portée sur le critère de jugement principal mais également sur les critères de jugement secondaire après vérification préalable sur la qualité et l'exhaustivité des données recueillies.

13.4. AUDIT ET INSPECTION

Un audit peut être réalisé à tout moment par des personnes mandatées par le promoteur et indépendantes des personnes menant la recherche. Il a pour objectif de vérifier la sécurité des participants et le respect de leurs droits, le respect de la réglementation applicable et la fiabilité des données s

Une inspection peut également être diligentée par une autorité compétente (ANSM pour la France ou EMA dans le cadre d'un essai européen par exemple).

L'audit, aussi bien que l'inspection, pourront s'appliquer à tous les stades de la recherche, du développement du protocole à la publication des résultats et au classement des données utilisées ou produites dans le cadre de la recherche.

Les investigateurs acceptent de se conformer aux exigences du promoteur en ce qui concerne un audit et à l'autorité compétente pour une inspection de la recherche.

14. CONSIDERATIONS ETHIQUES ET REGLEMENTAIRES

Le promoteur et l'(es) investigateur(s) s'engagent à ce que cette recherche soit réalisée en conformité avec la loi n°2012-300 du 5 mars 2012 relative aux recherches impliquant la personne humaine, ainsi qu'en accord avec les Bonnes Pratiques Cliniques (I.C.H. version 4 du 9 novembre 2016 et décision du 24 novembre 2006) et la déclaration d'Helsinki (qui peut être retrouvée dans sa version intégrale sur le site http://www.wma.net).

La recherche est conduite conformément au présent protocole. Hormis dans les situations d'urgence nécessitant la mise en place d'actes thérapeutiques précis, l'(es) investigateur(s) s'engage(nt) à respecter le protocole en tous points en particulier en ce qui concerne le recueil du consentement et la notification et le suivi des événements indésirables graves.

Cette recherche a reçu l'avis favorable du Comité de Protection des Personnes (CPP) d'Ile de France 1.

Le CHU de Toulouse, promoteur de cette recherche, a souscrit un contrat d'assurance en responsabilité civile auprès de Lloyds assurance conformément aux dispositions du code de la santé publique.

Les données enregistrées à l'occasion de cette recherche font l'objet d'un traitement informatisé à CHU de Toulouse dans le respect de la loi n°78-17 du 6 janvier 1978 modifiée par loi n°2018-493 du 20 juin 2018 relative à l'informatique, aux fichiers et aux libertés ainsi que du Règlement Général sur la Protection des Données, règlement n° 2016/679 adopté par le Parlement Européen le 16 avril 2016.

Cette recherche entre dans le cadre de la « Méthodologie de référence » (MR-001) en application des dispositions de l'article 54 alinéa 5 de la loi du 6 janvier 1978 modifiée relative à l'information, aux fichiers et aux libertés. Ce changement a été homologué par décision du 5 janvier 2006, mise à jour le 21 juillet 2016. Le CHU de Toulouse a signé un engagement de conformité à cette « Méthodologie de référence ».

- Cette recherche est enregistrée sur le site http://clinicaltrials.gov/

MODIFICATIONS AU PROTOCOLE

Toute modification substantielle, c'est à dire toute modification de nature à avoir un impact significatif sur la protection des personnes, sur les conditions de validité et sur les résultats de la recherche, sur la qualité et la sécurité des produits expérimentés, sur l'interprétation des documents scientifiques qui viennent appuyer le déroulement de la recherche ou sur les modalités de conduite de celle-ci, fait l'objet d'un amendement écrit qui est soumis au promoteur ; celui-ci doit obtenir, préalablement à sa mise en œuvre, un avis favorable du CPP et, le cas échéant, une autorisation de l'ANSM.

Les modifications non substantielles, c'est à dire celles n'ayant pas d'impact significatif sur quelque aspect de la recherche que ce soit, sont communiquées au CPP à titre d'information.

Toutes les modifications sont validées par le promoteur, et par tous les intervenants de la recherche concernés par la modification, avant soumission au CPP et, le cas échéant, à l'ANSM. Cette validation peut nécessiter la réunion de tout comité constitué pour la recherche. .

Toutes les modifications au protocole doivent être portées à la connaissance de tous les investigateurs qui participent à la recherche. Les investigateurs s'engagent à en respecter le contenu.

Toute modification qui modifie la prise en charge des participants ou les bénéfices, risques et contraintes de la recherche fait l'objet d'une nouvelle note d'information et d'un nouveau formulaire de consentement dont le recueil suit la même procédure que celle précitée.

15. CONSERVATION DES DOCUMENTS ET DES DONNEES RELATIFS A LA RECHERCHE

Les documents suivants relatifs à cette recherche sont archivés par l'investigateur conformément aux Bonnes Pratiques Cliniques :

***- pour une durée de 15 ans suivant la fin de la recherche** :*

- Le protocole et les modifications éventuelles au protocole
- Les cahiers d'observation (copies)
- Les dossiers source des participants ayant signé un consentement
- Tous les autres documents et courriers relatifs à la recherche
- L'exemplaire original des consentements éclairés signés des participants

Tous ces documents sont sous la responsabilité de l'investigateur pendant la durée réglementaire d'archivage. Aucun déplacement ou destruction ne pourra être effectué sans l'accord du promoteur. Au terme de la durée réglementaire d'archivage, le promoteur sera consulté pour destruction. Toutes les données, tous les documents et rapports pourront faire l'objet d'audit ou d'inspection.

16. RAPPORT FINAL

Dans un délai d'un an suivant la fin de la recherche ou son interruption, un rapport final sera établi et signé par le promoteur et l'investigateur. Ce rapport sera tenu à la disposition de l'autorité compétente. Le promoteur transmettra au CPP et, le cas échéant, à l'ANSM les résultats de la recherche sous forme d'un résumé du rapport final dans un délai d'un an après la fin de la recherche.

17. REGLES RELATIVES A LA PUBLICATION

17.1. COMMUNICATIONS SCIENTIFIQUES

L'analyse des données fournies par les centres investigateurs est réalisée par le CHU de Toulouse. Cette analyse donne lieu à un rapport écrit qui est soumis au promoteur, qui transmettra au Comité de Protection des Personnes et à l'autorité compétente.

Toute communication écrite ou orale des résultats de la recherche doit recevoir l'accord préalable de l'investigateur coordonnateur et, le cas échéant, de tout comité constitué pour la recherche.

L'investigateur coordonnateur/principal s'engage à mettre à disposition du public les résultats de la recherche aussi bien négatifs et non concluants que positifs.

La publication des résultats principaux mentionne le nom du promoteur, de tous les investigateurs ayant inclus ou suivi des participants dans la recherche, des méthodologistes, biostatisticiens et data managers ayant participé à la recherche, des vigilants ayant participé à l'analyse de la sécurité des participants, des membres du(des) comité(s) constitué(s) pour la recherche et la source de financement. Il sera tenu compte des règles internationales d'écriture et de publication (*The Uniform Requirements for Manuscripts* de l'ICMJE, avril 2010).

17.2. COMMUNICATION DES RESULTATS AUX PARTICIPANTS

Conformément à la loi n°2002-303 du 4 mars 2002, les participants sont informés, à leur demande, des résultats globaux de la recherche.

17.3. CESSION DES DONNEES

La gestion des données est assurée par le CHU de Toulouse. Les conditions de cession de tout ou partie de la base de données de la recherche sont décidées par le promoteur de la recherche et font l'objet d'un contrat écrit.

RÉFÉRENCES BIBLIOGRAPHIQUES

1. Chella A, Pipitone A. A cognitive architecture for inner speech. Cognitive Systems Research. 2020 Jan;59:287–92.
2. Di Nuovo A, Varrasi S, Lucas A, Conti D, McNamara J, Soranzo A. Assessment of Cognitive skills via Human-robot Interaction and Cloud Computing. Journal of Bionic Engineering. Springer Singapore; 2019 May;16(3):526–39.
3. Tanioka R, Locsin R, Yasuhara Y, Tanioka T. Potential Legal Issues and Care Implications during Care-Prevention Gymnastic Exercises for the Elderly Using Pepper in Long Term Health Care Facilities. Intelligent Control and Automation. Scientific Research Publishing; 2018 Aug 24;09(03):85–93.
4. Aaltonen I, Arvola A, Heikkilä P, Lammi H. Hello Pepper, May I Tickle You? New York, New York, USA: ACM Press; 2017. pp. 53–4.
5. Schicchi D, Pilato G. A Social Humanoid Robot as a Playfellow for Vocabulary Enhancement. IEEE; pp. 205–8.
6. Schicchi D, International GP2SI, 2018. A Social Humanoid Robot as a Playfellow for Vocabulary Enhancement - IEEE Conference Publication. ieeexploreieeeorg
7. Piezzo C, Suzuki K. Design of an accompanying humanoid as a walking trainer for the elderly. IEEE; pp. 467–72.
8. Tanaka F, Isshiki K, Takahashi F, Uekusa M, Sei R, Hayashi K. Pepper learns together with children: Development of an educational application. IEEE; pp. 270–5.
9. Pandey AK, Gelin R, Automation ARIR, 2018. Pepper: The First Machine of Its Kind. Researchgatenet
10. Diehl J, Schmitt L, Villano M, Crowell C. The clinical use of robots for individuals with autism spectrum disorders: a critical review. Research in Autism Spectrum Disorders; 6 (2012) 249–26211. Ismail L, Shamsudina S, Yussofa H, Hanapiahc F, Zaharid N. Robot-based Intervention Program for Autistic Children with Humanoid Robot NAO: Initial Response in Stereotyped Behavior ; Procedia Engineering 41 (2012) 1441 – 1447

Références bibliographique des questionnaires standardisés

[1] Agarwal, R., & Karahanna, E. (2000). Time flies when youare having fun: Cognitive absorption and beliefs about IT usage. MIS Quarterly, 24(4), 665–694. doi:10.2307/3250951
-[7] Desmet, P.M.A., Vastenburg, M.H., Romero Herrera, N. (2016). Mood measurement with Pick-A)Mood : review of current methods and design in a pictorial self-report scale. J. of Design Research, 14(3), 241-279
[9] Heerink, M., Krose, B., Evers, V., & Wielinga, B. (2010). Assessing acceptance of assistive social agent technology by older adults: The Almere Model. International Journal of Social Robotics, 2(4), 361–375.
[12] Lallemand, C., Gronier, G., (2018). Méthodes de design UX : 30 méthodes fondamentales pour concevoir des expériences optimale. Eyrolles.
[13] Lallemand, C. & Koenig, V. (2017). "How Could an Intranet be Like a Friend to Me?" – Why Standardized UX Scales Donat Always Fit. Proceedings of ECCE 2017, Umea, Sweden.
[14] Lallemand, C., Koenig, V., Gronier, G., & Mar5n, R. (2015). Création et validation d'une version française du questionnaire AGrakDif pour l'évaluation de l'expérience utilisateur des systèmes interactifs, Revue Européenne de Psychologie Appliquée. doi:10.1016/j.erap.2015.08.002 [16] Moon, J. W., & Kim, J. G. (2000). Extending the TAM for a world-wide-web context. Information & Management, 38, 217–230.
[17] Read, J., MacFarlane, S., Casey, C., (2002) Endurability, Engagement and Expectations: Measuring Childrenas Fun
[15] Sánchez-Morales, A., Durand-Rivera, J. A., & Martínez-González, C. L. (2020). Usability evaluation of atangible user interface and serious game for identification of cognitive deficiencies in preschool children. *International Journal of Advanced Computer Science and Applications, 11*(6), 486-493.
[16] Xu, D., Read, J. C., Sim, G., & McManus, B. (2009, June). Experience it, draw it, rate it: capture children's experiences with their drawings. In Proceedings of the 8th International Conference on Interaction Design and Children (pp. 266-270)

Evaluation of the acceptability of a humanoid robot in the home for the evaluation of children with cochlear implants

H2R2

RESEARCH PROTOCOL RESEARCH INTERVENTIONAL RESEARCH IMPLIQUANT THE HUMAN PERSON ***category 2 with minimal risks and constraints***

Version n°3.*0 of 04/07/2022*

Sponsor Code: RC31/20/0250

BCID Number: 2020-A01989-30.

This interventional research has obtained funding from the Occitanie region - "Research & Society 2018" call for projects.

Sponsor:
CHU TOULOUSE - Hôtel Dieu - 2, rue Viguerie - TSA 80035 31059 Toulouse cedex 9

Principal investigator (single-center research):
Pr DEGUINE Olivier
PU-PH
Toulouse University Hospital - Pierre Paul Riquet
ENT, Pediatric ENT and Otoneurology Department
Place du Dr Baylac
31059 Toulouse

This protocol was designed and written based on the GIRCI SOHO model protocol version 5.0 dated 10/30/2017

HISTORY OF UPDATES TO THE PROTOCOL

V ERSION	DATE	RATIONALE FOR UPDATE
1	29/01/2020	Request for promotion at the TOULOUSE University Hospital
1.1	02/07/2020	Initial submission to CPP Sud-Med II
1.2	23/11/2020	Initial submission to IDF PPC 1
1.3	05/02/2021	Response to IDF PPC comments 1
2.0	28/10/2021	Substantial Amendment No. 1
3.0	04/07/2022	Substantial Amendment No. 2

SIGNATURE PAGE OF THE PROTOCOLE

Evaluation of the acceptability of a humanoid robot placed in the home of a cochlear implanted child

H 2R2

Sponsor Code: RC 31/20 -0250

**Developer**<br><br>TOULOUSE University Hospital<br>Hotel Dieu<br>2, rue Viguerie TSA 80035<br>31059 TOULOUSE cedex 9<br>Tel : +33 (0)5 61 77 86 03<br>Fax : +33 (0)5 61 77 84 11<br>*drci.toulouse@chu-toulouse.fr*	Done in<br><br>Toulouse, On	Mr Olivier LAIREZ ,<br>Director of Research and Innovation<br>*signature*
**Principal Investigator**<br><br>*Pr DEGUINE Olivier*<br>*PU-PH*<br>*CHU Toulouse - Pierre Paul Riquet*<br>*ENT, Pediatric ENT and Otoneurology Department*<br>*Place du Dr Baylac*<br>*31059 Toulouse*<br>Email: *deguine.o@chu-toulouse.fr*	Done in Toulouse<br><br>On	Pr DEGUINE Olivier<br>PU-PH<br><br>*signature*

MAIN CORRESPONDENTS

Principal Investigator
Pr DEGUINE Olivier
CHU Toulouse - Pierre Paul Riquet ENT, Pediatric ENT and Otoneurology Department
Place du Dr Baylac
31059 Toulouse

Associate Investigator
Dr Calmels Marie Noelle
ENT, Ear, Nose and Throat and Pediatric ENT Department
Pierre Paul Riquet Hospital
Place du Dr Baylac
31059 Toulouse
Tel : 05 61 77 90 09

Dr. Baladi Blandine
ENT, Ear, Nose and Throat and Pediatric ENT Department
Pierre Paul Riquet Hospital
Place du Dr Baylac
31059 Toulouse

Pr Marx Mathieu
ENT, Ear, Nose and Throat and Pediatric ENT Department
Pierre Paul Riquet Hospital
Place du Dr Baylac
31059 Toulouse

Dr. Yohan Gallois
ENT, Ear, Nose and Throat and Pediatric ENT Department
Pierre Paul Riquet Hospital
Place du Dr Baylac
31059 Toulouse

Other specialties
Cochard Nadine (speech therapist) Husson Hélène (speech therapist)
Lasfargues Anne (speech therapist)
Pierre Paul Riquet Hospital
Place du Dr Baylac
31059 Toulouse

Clinical Research Vigilance Unit
Dr Pascale OLIVIER -ABBAL
Department of Medical and Clinical Pharmacology & Research Directorate Development and Innovation
CHU TOULOUSE
Tel : 05 61 14 59 98 (CRPV)
Tel : 05 61 77 85 56 (DRDI)

Maison des Sciences de l'Homme et de la société
Pierre Vincent Paubel
University Toulouse Jean Jaurès
5 Allée Antonio Machado
31058 Toulouse cedex 9

Cognition, Language, Ergonomics Laboratory - UMR 5263 - CNRS
Pascal Gaillard
Loïc Caroux
Maison de la Recherche
Université Toulouse Jean Jaurès
5 Allée Antonio Machado
31058 Toulouse cedex 9

Brain and Cognition Research Center - UMR 5549
Pascal Barone
Kuzma Streilnikov
Chloe Farrer
Sabrina STITI
Pavillon Baudot
Purpan
31059 Toulouse

Project support
Institute for Advanced and Surgical Technologies (ITAC)
Project manager : Aline MEULLE
CHU Toulouse - Pierre Paul Riquet Hospital
Hall C - 2nd floor
Place du Dr Baylac
31059 Toulouse

Developer
CHU TOULOUSE - Hotel
Dieu - 2, rue Viguerie - TSA 80035
31059 Toulouse cedex 9

Research manager: Dr
Marie-Elise LLAU
Tel : 05 61 77 86 03

Regulatory CRA : Florine LEGAY

Methodology Center
Clinical Research Support Unit (USMR)
Pharmacology Department - Faculty of
Medicine - 37 allées Jules Guesde
31000 Toulouse

Methodologist: Benoit Lepage

SUMMARY

Protocol signature page 3
SUMMARY **6**
1. RESEARCH SUMMARY **9**
2. SCIENTIFIC JUSTIFICATION AND GENERAL DESCRIPTION **12**
2.1. CURRENT STATE OF KNOWLEDGE 12
2.1.1. On the pathology 12
2.1.2. On the treatments/strategies/procedures underconsideration 12
2.2. RESEARCH HYPOTHESES AND EXPECTED RESULTS 12
2.3. JUSTIFICATION OF THE METHODOLOGICAL CHOICES 12
2.4. PROFIT / RISK RATIO 12
2.5. EXPECTED BENEFITS 13
2.6. RATIONALE FOR THE LEVEL OF INTERVENTION 13
3. OBJECTIVES OF THE RESEARCH 14
3.1. MAIN OBJECTIVE 14
3.2. SECONDARY OBJECTIVES 14
4. JUDGING CRITERIA **14**
4.1. PRIMARY ENDPOINT 14
4.2. SECONDARY ENDPOINTS 14
DESIGN OF THE RESEARCH **16**
4.3. RESEARCH SCHEME 16
5. ELIGIBILITY CRITERIA **16**
5.1. INCLUSION CRITERIA 16
NON-INCLUSION CRITERIA 16
5.2. FEASIBILITY AND RECRUITMENT MODALITIES 16
6. TREATMENT(S)/STRATE RESEARCH METHOD(S)/PROCEDURE(S) 17
6.1. EXPERIMENTAL TREATMENT/STRATEGY/PROCEDURE 17
7. TREATMENTS AND ASSOCIATED PROCEDURES
7.1. ASSOCIATED TREATMENTS/PROCEDURES ALLOWED 17
7.2. ASSOCIATED PROHIBITED TREATMENTS/PROCEDURES 17
8. HOW THE RESEARCH IS CONDUCTED SEARCH 18
8.1. RESEARCH SCHEDULE 18
8.2. SUMMARY TABLE OF THE PARTICIPANT FOLLOW-UP 18
8.3. PRE-INCLUSION & INCLUSION VISIT 19
8.3.1. Collection of consent 19
8.3.2. Conduct of the Inclusion Visit 19
8.4. FOLLOW-UP HOME VISITS D21, D28, D36 19
8.5. END OF RESEARCH VISIT 20
8.6. RULES FOR TERMINATION OF A PERSON'S PARTICIPATION IN RESEARCH 20
8.7. RESEARCH CONSTRAINTS AND POSSIBLE COMPENSATION OF PARTICIPANTS

9. MANAGEMENT OF ADVERSE EVENTS/INCIDENTS **21**
10. STATISTICAL ASPECTS **21**
10.1. CALCULATION OF THE STUDY SIZE 21
10.2. STATISTICAL METHODS USED 21
11. MONITORING OF R ECHERCHE **22**
12. ACCESS RIGHTS TO DATA AND DOCUMENTS SOURCE **22**
12.1. ACCESS TO DATA 22
12.2. SOURCE DATA 22
12.3. CONFIDENTIALITY OF DATA 22
13. QUALITY CONTROL AND ASSURANCE **23**
13.1. GUIDELINES FOR DATA COLLECTION 23
13.2. QUALITY CONTROL 23
13.3. DATA MANAGEMENT 23
13.4. AUDIT AND INSPECTION 24
14. ETHICAL AND REGULATORY CONSIDERATIONS NICAL AND REGULATORY CONSIDERATIONS S **24**
15. RETENTION OF RESEARCH AND DATA RELATED TO THE ERCHE RESEARCH. **25**
16. FINAL REPORT **25**
17. PUBLICATION RULES **25**
17.1. SCIENTIFIC COMMUNICATIONS 25
17.2. COMMUNICATION OF RESULTS TO PARTICIPANTS 26
17.3. TRANSFER OF DATA 26
REFERENCES BIBLIOGRAPHICS **27**

L ISTE OF ABREVIATIO NS

ANSM	National Agency for the Safety of Medicines and Health Products
CPP	Committee for the Protection of Individuals
EvI	Undesirable event
EvIG	Serious Adverse Event
SAE	Serious Adverse Effect
SAE	Unexpected Serious Adverse Effect
SUSAR	Suspected Unexpected Serious Adverse Reaction

1. SUMMARY OF THE RESEARCH

**DEVELOPER**	Toulouse University Hospital
**INVESTIGATOR COORDINATOR/PRINCIPAL**	Pr DEGUINE Olivier<br>Toulouse University Hospital - Pierre Paul Riquet<br>ENT, Pediatric ENT and Otoneurology Department<br>Place du Dr Baylac<br>31059 Toulouse
**T ITRE**	Evaluation of the acceptability of a humanoid robot placed in the home of a cochlear implanted child -H2R2
**J USTIFICATION / CONTEXT**	The quality of the rehabilitation of the deaf child with a cochlear implant is a major prognostic factor in the outcome of speech understanding and expression. This rehabilitation is performed jointly by the cochlear implant hospital team and a speech therapist located near the child's home. The multi-weekly sessions represent a constraint for the child and his/her parents due to the necessary availability, travel, and anxiety generated by the non-ecological environment in the office or hospital. In addition, some territories are insufficiently provided with speech therapists, making access to care more difficult. A complementary training work at home would allow to balance the equity of distribution of care in the territory, and should favour the progress of the child, more inclined to use a tool available at home. The humanoid robot should allow an ecological approach of this rehabilitation complement. Before developing this approach, it is necessary to study the acceptability of the humanoid robot in home, both by the parents and by the cochlear implanted child.
**OBJECTIVES**	The main objective is to evaluate the acceptability by the child and his family of a humanoid robot installed at home for one month.<br><br>The secondary objectives are<br>- to describe the acceptability of the child and his family with respect to the relationship with the technology, the intention of use, the expectations, the perceived usefulness, the perception of the robot and the facilitating conditions before and after 1 month of use of the robot at home,<br>- describe the child's acceptability of his or her experience, enjoyment, and feelings when using the robot at home
**C RITERIA OF JUDGEMENT**	**Primary endpoints**<br>The acceptability of a humanoid robot at home, by the child and his family, will be evaluated by the number of hours per week during which the child solicits the robot, at home.<br><br>**Secondary endpoints**<br>The criteria for the secondary objectives are as follows<br><br>• Responses to standardized questionnaires and scales<br>• Sentence completion<br>• The "pick-a-mood" version of emotion. Categorization and classification of activities<br>• Semi-structured interview

**SCHEME OF THE RESEARCHE**	Prospective single-center open-label study evaluating the acceptability of a humanoid robot PEPPER in the homes of deaf children with cochlear implants.
**INCLUSION CRITERIA**	- Child aged 8 to 12 years old, user of his cochlear implant with a favorable family environment,<br>-Children implanted with at least one cochlear implant undergoing speech rehabilitation, and followed by the Pediatric Cochlear Implant Unit (UPIC) of the Toulouse University Hospital<br>- Child and family whose first language is French<br>-Affiliation to a social security scheme
**C RITERIA OF NON-INCLUSION**	- Cognitive or psychological incapacity or refusal of the participant to give written consent<br>- Other sensory or motor deficits that may interfere with the use of the robot<br>- Unstable psychiatric pathology<br>- Child whose parents a r e both benefiting from a measure of legal protection
**T RAITMENTS /STRATEGIES /PR OCEDURES OF RESEARCH RCHE**	Pepper is a humanoid robot measuring 1.20 m high. It is perfectly adapted to the use by children because it has the size of a human of 8 years.<br><br>For the study, we have two PEPPER robots that will have been previously programmed and implemented with the study tests. The robots will be put at the disposal of the patients in an alternating way. To date, the 2 robots have been implemented with the following elements:<br>• People recognition: facial identification<br>• Personalization for the child<br>• Personalization at the family's pace<br>• Activities<br>• Interaction<br><br>No data other than those necessary to meet the objectives of the study will be recorded or saved. Moreover, the robot has no remote connection and is not linked to the internet. No data can be transmitted remotely. The data will be retrieved by USB key during the monitoring visits.<br><br>Once the robot is available to the child at home, the child will be able to use it as many times as he or she wants and at any time. time of day
**T AIL L E OF STUDY**	N=10
**DURATION OF THE RESEARCH**	Duration of the inclusion period: 18 months<br>Actual duration of participation for each participant: 1 month Total duration of participation for each participant: approximately 1 year and 1 month<br>Total duration of the research (duration of the inclusion period + duration of participation): 20 months
**STATISTICAL ANALYSIS OF DATA**	A descriptive analysis of the endpoints will be performed. A linear mixed model will be applied to estimate the average time evolution of the number of hours weekly solicitation of the robot

**EXPECTED RESULTS**	Our project proposes to implement an interactive, personalized solution to help cochlear implanted children progress in language expression and comprehension by to complement the usual speech therapy. This

H2R2
Version n° 3.0 of 04/07/2022

	This first step will provide essential data to improve knowledge in the field of home acceptability of a humanoid robot. Eventually, the development of a humanoid robot adapted to the rehabilitation of deaf and implanted children at home will represent a therapeutic and technological breakthrough, since such a device could improve the quality of life and autonomy of these patients, and promote rehabilitation, especially in remote areas.

2. SCIENTIFIC JUSTIFICATION AND GENERAL DESCRIPTION

2.1. CURRENT STATE OF KNOWLEDGE AISSANCES

2.1.1. ON THE PATHOLOGY

Cochlear implantation is the reference treatment for restoring hearing and language development in children with total bilateral deafness that cannot be fitted with a hearing aid. It requires a surgical procedure, the indication of which is determined by multidisciplinary consultation. Post-operative speech rehabilitation and family involvement in this rehabilitation are major prognostic factors for speech understanding and language development after cochlear implantation.

2.1.2. ON THE TREATMENTS/STRATEGIES/PROCEDURES OF REFERENCE AND UNDER STUDY

Rehabilitation after cochlear implantation is organized in a coordinated manner between the pediatric cochlear implantation hospital team (UPIC), and the relay provided by a speech therapist located near the child's home. It involves multi-weekly auditory and speech training sessions. Regular visits to the implant center are necessary to verify the condition and proper use of the implant, to check the settings and to perform a speech therapy assessment. This assessment allows us to evaluate the child's progress in terms of understanding and expressing speech, and to adapt and guide the rehabilitation to the child's abilities.

This speech therapy rehabilitation can be limited by the constraints it imposes on families. These constraints are linked to the need for a high level of availability to accompany the child to the speech therapist or to the hospital, and to repeated transportation. In some cases, the geographical distance in under-medicalized areas can amplify these constraints. In addition, fatigue, time constraints, and stress related to the medical or paramedical environment can have a negative effect on the implanted child.

The use of a humanoid robot of the NAO type has been used successfully with children with autistic disorders as described by Diehl et al and Ismail et al.

The Pepper robot has been little used in care structures. It has shown its usefulness for the reception of people in commercial or industrial structures. Its reassuring aspect, its facial recognition and its morphology contribute to make it acceptable by people not initiated to the robotics approach.

2.2. HYPOTHESES OF THE RESEARCH AND EXPECTED RESULTSEXPECTED

We postulate that the humanoid robot PEPPER used at home will be well accepted by the deaf child and his family.

2.3. JUSTIFICATION OF CHOICES METHODOLOGICAL

Research design: At this very early stage of the evaluation of the use of the Pepper robot in the home, we propose a pilot study on a small number of patients, sufficient to meet acceptability evaluation criteria.

Choice of main objective: The acceptability of a humanoid robot in the home is a prerequisite for the implementation of a rehabilitation aid for deaf children with cochlear implants.

Choice of population: The children selected are part of the cohort regularly followed by UPIC physicians and speech therapists. They will be selected from among those who have an optimal use of the cochlear implant, with a favorable family environment.

2.4. PROFIT / LOSS RATIO ISK

INDIVIDUAL BENEFITS

The use of the robot at home by the child, at any time of the day and as many times as the child wishes, reduces the constraints related to rehabilitation in the office or in the hospital. The proposal of "educational" games oriented on language allows the child to be freed from the stress of speech

therapy sessions, while respecting his life rhythm. It is a personalized rehabilitation in a natural environment, which is difficult to achieve with the usual methods; it is hoped that the child will benefit in terms of comprehension andoral expression.

This is a typical example of personalized and participatory medicine, at the service of the patient and his family.

COLLECTIVE BENEFITS

This study will provide essential data on the acceptability of using humanoid robots in the home for children with cochlear implants. Demonstrating the acceptability of the use of robotics in the home will be a major advance in facilitating the care pathway and reducing territorial inequalities in under-medicalized areas.
The results of this acceptability study could lead, in case of positive results, to the implementation of studies evaluating the effectiveness of the use of a humanoid robot in the rehabilitation of cochlear implant patients and more generally of patients with disabilities. This information will be particularly useful for people with reduced mobility.

RISKS

It is a commercialized CE marked robot, commonly used for human/robot interactions. Its use falls within the scope of its design. There is no known risk of using this tool.

CONTRAINTS

The constraints related to the research are considered minimal. Participation in the study will require 4 home visits of approximately 1 hour - 1.5 hours by the investigator or his representative; the constraint is therefore related to the availability of the family during this visit. The child will be free to use the robot whenever he/she wishes, and for as long as he/she wishes.
In addition, the child and his/her parents will be asked to complete various questionnaires during the visits. The time to complete these questionnaires varies between 20 and 75 minutes.

The benefit/risk ratio can therefore be considered as very favorable.

2.5. BENEFITS EXPECTED

Our project proposes to implement an interactive, personalized solution to help cochlear implanted children progress in the development of understood and spoken language, in addition to the usual speech therapy. This first step will provide essential data to improve knowledge in the field of the acceptability of a humanoid robot at home. Eventually, the development of a humanoid robot adapted to the rehabilitation of deaf and implanted children at home will represent a therapeutic and technological evolution, by making a rehabilitation device available in an ecological environment (the home), which can be used without limit, at the rhythm chosen by the child and his family.

2.6. JUSTIFICATION FOR THE LOW LEVEL OF INTERVENTION

This can be considered a research protocol generating minimal risks and constraints for the patient (category 2 RIPH), in accordance with the order of April 12, 2018 establishing the list of research with minimal risks and constraints :
"2. Administration or use of products placed on the market within the European Union, when the conditions of use of these products are in accordance with their intended purpose and their current conditions of use.
11. Interviews, observations and questionnaires whose results, in accordance with the protocol, may lead to a change in the participant's usual medical care and which are not therefore part of the research mentioned in 3° of article L. 1121-1 of the public health code.

The time constraint in relation to the research protocol corresponds to 1h - 1h30 of home interviews, every week for one month, as well as the time to complete the questionnaires (from 20 to 75 minutes depending on the visit)

The risks related to the research protocol can be considered as minimal, and correspond to the risks of using the robot. The robot, CE marked, is used to perform speech therapy tests and games with the child. The conditions of use of the robot in the project are consistent with its current use since the Pepper robot is designed to interact with humans.

3. OBJECTIVES OF THE RESEARCH

3.1. OBJECTIVE

The main objective is to evaluate the acceptability by the child and his family of a humanoid robot placed for one month at home.

3.2. SECONDARY OBJECTIVES

The secondary objectives are

- to describe the acceptability of the child and his family with respect to the relationship to the technology, the intention of use, the expectations, the perceived usefulness, the perception of the robot and the facilitating conditions before and after one month of use of the robot at home,
- describe the acceptability of the child's experience, enjoyment and emotions when using the robot at home

4. CRITERIA OF JUDGMENT

4.1. JUDGEMENT CRITERION MAIN

The acceptability of a humanoid robot at home, by the child and his family, will be evaluated by the number of hours per week during which the child solicits the robot.

Each time the robot is used, the time of use will be recorded.

4.2. JUDGING CRITERIA SECONDARY

The secondary objective endpoints are:

- Standardized questionnaires and scales validated by the scientific community evaluate the relationship with the technology, the intention of use, the relationship with the robot and the user experience. They allow to compare the answers of different participants between them, but also those of the same participant at two given moments. Sentence completion is used to collect participants' expectations. This is a technique used in the field of UX (user experience) which consists of giving the user the beginning of a sentence and letting them complete it freely. This allows us to have more subjective data and to complement the data obtained through questionnaires. The children will take the SUS (Systeme Usability Scale) which is a standardized and validated UX questionnaire that measures the usability of the robot by the child. The version used was adapted for children by Sanchez-Morales et al.,2020 [(15)]. It consists of 10 affirmative sentences. An analysis of the drawings made by the child will be performed using a drawing scoring grid (drawing method of XU ET AL, 2009) [16].
- A "pick-a-mood" version of the child's emotion (girl/boy), adapted from the "pick-a-mood" version The robot's "perceived emotion" will help assess the child's emotions.
- The categorization and classification of the activities by the children: done according to various criteria (good functioning, fun, ease, among others) it allows to have a feedback on the content implemented on the robot.
- The semi-directive interview: planned as the last collection of this experience, it should allow to obtain a more detailed and free feedback from the parents, but also from the implanted child. The parents and the child will participate in a separate interview when the robot is uninstalled.

All the questionnaires and methods used are detailed in the table below:

**Questionnaires standardisés**		
**Attrakdif [14]**	Mesure du rapport à la technologie en général	Parents
**Personal innovativness [1]**	Rapport à la technologie	Parents/Enfant
**Use Intention Scale [16]**	Intention d'usage	Parents/Enfant
	Utilisation effective	Enfant
**Heerink questionnaire [9]**	Utilité perçue, anxiété, attitude d'usage, conditions facilitatrices, intention d'usage, adaptivité perçue, appréciation perçue, facilité d'utilisation perçue, sociabilité perçue, influence sociale, présence sociale, confiance	Parents
**MeCUE [13]**	Expérience utilisateur, intention d'usage, utilité perçue,	Enfant
SUS [15]	utilisabilité, influence sociale, satisfaction	Child
**Echelles**		
**Fun-o-meter [17]**	Amusement	Enfant
**Pick-a-mood [7]**	Emotions perçue du robot	Enfant
**Autres méthodes**		
**Complétion de phrases [12]**	Attentes, attitude	Parents/Enfant
**Classement des activités [17]**	Amusement, satisfaction, préférences dans les activités	Enfant
**Catégorisation des activités [17]**	Intention d'usage spécifique pour les activités	Enfant
**Entretien**	Relation robot/enfant, recueil de subjectivité	Parents/Enfant
**Recueil d'informations**	Expérience passée, rapport à la technologie	Parents/Enfant

DESIGN OF THE RESEARCH

4.3. RESEARCH DESIGN

This is a single-center, prospective, open-label pilot study to evaluate the acceptability of a humanoid robot PEPPER in the homes of children with cochlear implants.

5. ELIGIBILITY CRITERIA

5.1. INCLUSION CRITERIA

Inclusion criteria were as follows:

- Child aged 8 to 12 years, user of his cochlear implant with a favorable family environment,
- Children implanted with at least one cochlear implant and undergoing speech rehabilitation, and followed by the Pediatric Cochlear Implant Unit (UPIC) of the Toulouse University Hospital
- Child and family whose first language is French
- Affiliation to a social security scheme

CRITERIA FOR NON-INCLUSION USION

The exclusion criteria are as follows:

- Cognitive or psychological incapacity or refusal of the participant to give written consent
- Other sensory or motor deficits that may interfere with the use of the robot
- Unstable psychiatric pathology
- Child whose parents are under legal protection

5.2. FEASIBILITY AND MODALITIES OF RECRUTEMENT

The children were recruited in the ENT, Otoneurology and Pediatric ENT departments of the Toulouse University Hospital during a consultation as part of their speech therapy follow-up.

This service is specialized in the management of pediatric deafness and has extensive experience in clinical research. The active file is 410 cochlear implanted children, in constant progression (28 per year in 2015, 39 per year in 2019), which is more than sufficient to ensure the recruitment of the 10 patients for our feasibility study.

In the framework of this research protocol, only patients already present in the active fi le of routine care are recruited, which facilitates recruitment and limits the number of losses of sight. The number of lost to follow-up should also be limited because of the protocol which provides a robot at the patient's home: this provision implies regular monitoring of the proper use of the robot by the project team. In this context, non-identifying photos of the robot's installation environment will be taken and the company installing the robot will provide information on certain parameters such as the presence of pets or the layout of the robot.

In practice, the investigators propose to children and their parents/legal guardians who meet all the inclusion criteria, without any non-inclusion criteria, to participate in the H2R2 research protocol.

The patient pathway corresponds to that of usual care:

- The first visit is the inclusion visit and allows the patient to become familiar with the robot
- The robot is installed at the child's home for 1 month depending on the availability of the parents and the robot. The child can interact with it as many times as he/she wants. During this month, the investigator or his representative will visit the patient's home every week to make sure that the robot is being used properly and to collect the difficulties encountered and the experience of the family and the child. Coverage for speech therapy remains unchanged during the period of the child's participation in the study.

6. TREATMENT(S)/STRATEGY(IES)/PROCEDURE(S) OF THE RESEARCH

6.1. TREATMENT/STAGE MSE/PROCEDURE EXPERIMENTAL

Pepper is a humanoid robot measuring 1.20 m high. It is perfectly adapted to the use by children because it has the size of a human of 8 years. It has 17 joints that allow a body language, 3 omnidirectional wheels to move easily. It reaches the maximum average walking speed of a human. It has a touch pad, allowing manual interaction to complement the vocal interaction. The robot speaks, hears, and has a facial identification.
It has an autonomy of more than 12 hours of energy for continuous activities. The tablet is compatible with a wide range of content: videos, applications, texts, buttons, etc. This accessory becomes a real additional interface to interact with the Pepper robot.

For the study, we have two PEPPER robots that will have been previously programmed to meet the needs of the study. The robots will be made available to the patients on an alternating basis. Between each patient, the robot will remain at MSHS for one month to allow the team to prepare it for the next patient.

To date, the 2 robots have been implemented with the following elements:

- Person recognition: The robot registers the first name and face of family members and recognizes them. It addresses people using their first names and can register new faces if asked. The robot also distinguishes between the implanted child and others and offers activities only to the implanted child; however, everyone else can interact with it.
- Personalization for the child: The robot can ask a few questions about the interests identified by the child beforehand. It can also make remarks related to these topics during the interaction.
- Personalization to the family rhythm: the robot knows the child's bedtime and mealtime and does not propose any activity during these times, in order not to disturb the family rhythm. It also makes remarks related to these moments (e.g.: "I'm getting hungry" at noon).
- Activities: The robot offers 3 games (hangman, memory and animal riddles), a story activity to complete, dances and dances to imitate.
- Interaction: The robot can answer general questions about itself, temporality (day, time) and deafness. The robot can also tell some jokes. The robot offers the child a break after a certain amount of interaction and also asks if he is having fun.

Note that the robot does not use its microphone to record data in the home environment. No data other than that necessary to meet the objectives of the study will be recorded or saved. Furthermore, the robot has no remote connection and is not connected to the internet. No data can be transmitted. The collection of data useful to the project (time of use) will be recovered by USB key during monitoring visits.

These characteristics have been implemented in the robot by the engineers of the Maison des Sciences de l'Homme et de la Société and by the researchers of the CLLE laboratory.

Once the robot is at the child's disposal at home, the child will be able to use it as many times as he wants and at any time of the day.

7. ASSOCIATED TREATMENTS AND PROCEDURES

7.1. TREATMENTS/PROCEDURES ASSOCIATED TREATMENTS/PROCEDURES ISE(S)

Children will be able to continue to take their usual medication.

7.2. TREATMENTS/PROCEDURES ASSOCIATED TREATMENTS/PROCEDURES DIT(E)S

No treatment or procedure is allowed

8. PROCEDURE OF THE RESEARCH

8.1. research schedule

– Duration of the inclusion period: 18 months
– Effective duration of participation of each participant: 1 month
– Total duration of participation of each participant: 1 year and 1 month
– Total duration of the research: 20 months

8.2. SUMMARY TABLE OF FOLLOW-UP PARTICIPANT

	Pre-inclusion (0 to 15 days before V0)	Inclusion[1] V0	Visit 1 *V1 : J1 (installation n)*	Visit 2 *V2 : D8+2 After installation*	Visit 3 *V3 : D15+2 After installation*	Visit 4 *V4 : D22+2 After installation*	Visit 5 V5 : D29+2 *After installation*
	CONS.	CONS.	HOME				
InformationNotice*(R)*	✓	✓					
Informed consent *(R)*		✓					
Verification of eligibility criteria *(R)*	✓	✓					
Installation of the robot at home (R)			✓				
Uninstalling the robot (R)							✓
Preliminary evaluation of acceptability (relationship to the technology, intention ofuse, expectations, perceived usefulness, perception of the robot, facilitating conditions) in the child and his family (R)		✓					
Intermediate evaluation: Evaluation of the user experience, fun and emotionsfelt by the child (R)				✓	✓	✓	
Intermediate evaluation: Evaluation of theuser experience, the emotions felt by the parents (R)				✓		✓	
Final feedback: Evaluation of the user experience forchildren and their families (R)							✓
Collection of robot data on a USB key (date and time of use, user, user's mood, type of application launched)			✓	✓	✓	✓	✓
Use of the robot by the child			✓ (as many times as the child wants)				

[1]Estimated maximum time between the signature of the consent and the installation of the robot is 1 year +/- 2 months.
CONS consultation

8.3. PRE-INCLUSION & INCLUSION VISIT INCLUSION

As part of the follow-up of the profoundly deaf child (with a functional cochlear implant), the family is seen in consultation by an investigating physician in the ENT department at the Pierre Paul Riquet Hospital (PPR) at Purpan University Hospital. During this consultation and after having verified the eligibility of the child, the investigating physician will inform the family about the study and will propose to them to participate. The information leaflet of the study will be given to the families.

8.3.1. COLLECTION OF CONSENT

During the inclusion visit, one of the investigating physicians informs the child and the representatives of parental authority and answers all their questions concerning the objective, the nature of the constraints, the foreseeable risks and the expected benefits of the research. He also specifies the rights of the child in the context of interventional research involving the human being and verifies the eligibility criteria.

The investigating physician is responsible for obtaining written informed consent from the parent(s). The consent form must be signed prior to any procedure under the research protocol.

If parental consent is given, the parents and the investigator should write their full names, date and sign the consent form. Although only the permission of the parent(s) is required, the minor's refusal to participate will not be overridden.

In accordance with the Loi Jardé for category 2 research, it will be possible to collect the consent of only one holder of parental authority for deaf children, if the consent of the other holder cannot be collected within the time limit set by the research protocol. The different copies of the information note and consent form are then distributed as follows:
- A copy of the information note and signed consent is given to the holders of parental authority
- The original copy is kept by the investigating physician (even if the patient moves during the course of the research) in a safe place inaccessible to third parties.

8.3.2. CONDUCT OF THE INCLUSION VISIT

The inclusion visit is conducted by the investigating physician. Before any research-related examination, the investigator obtains the free, informed and written consent of the participant (or his legal representative, if applicable).

An initial evaluation of the relationship to the technology, the intention of use, the expectations, the perceived usefulness, the perception of the robot as well as the facilitating conditions is carried out before the interaction with it. These elements will be measured both in the implanted child and in his parents. The completion of these questionnaires will last between 20 and 30 minutes for the parents and between 40 and 50 minutes for the child.

The Pepper robot will then be presented to the child so that he/she can become familiar with its use. The investigator will explain to the child and his family how the robot works and the different interactions the child can have with it.

The robot will then be installed for 1 month at the child's home depending on the availability of the parents and the robot. A service company will be in charge of installing and uninstalling the robot at the patients' home. The robot will be transported in its carrying case and the service providers will have to make sure that it works properly. The providers will also be able to answer the patients' technical questions if needed.

The service company will be specialized in the health field and authorized to carry out this type of benefit. Providers will not have access to patient medical data.

8.4. FOLLOW-UP HOME VISITS D8+2, D15+2, D22+2, D29+2

The installation of the robot in the child's home is at D1.

Follow-up visits will take place every week at the child's home by an investigator or his representative (employees of the service company, authorized to carry out this type of mission). During this visit, the user experience, the fun and the emotions felt by the implanted child in relation to his use of the robot will be evaluated. The completion of the questionnaire by the child will last between 20 and 25 minutes. The user experience, the fun and the emotions felt by the parent will be evaluated at D8+2 and D22+2.

The completion of the questionnaire by the parent will take between 20 and 25 minutes. The correct use of the robot will also be verified.

The installation of the robot by the service company and its removal will be subject to the hygiene rules in force: wearing a mask, respecting the physical distance, disinfection of the robot.
Non-identifying photos of the robot installation environment will be taken

The robot will be uninstalled during the visit at D29+2.

8.5. END OF PROJECT VISIT SEARCH

The end of research visit corresponds to the uninstallation of the robot at D29+2 . It will take place at the child's home with an investigator or his representative. During this visit, a final evaluation of the relationship with the technology, the intention of use, the expectations, the perceived usefulness, the perception of the robot as well as the facilitating conditions is carried out after 1 month of use at home. These elements will be measured both in the implanted child and in his parents. A semi-directive interview will be performed in order to obtain a more detailed and free feedback from the parents and the child. The completion of the questionnaire and the interview will last between 45 and 55 minutes for the parents and between 65 and 75 minutes for the child.

8.6. RULES FOR STOPPING THE PARTICIPATION OF A PERSON TO RESEARCH

A person's participation in the research may be terminated in the following cases:

- Deliberate decision of the patient the participation of a patient in research will stop in case of withdrawal of consent;
- Due to the death of the person ;
- Subject is lost to follow-up: If the patient is no longer followed in the study and no cause is identified after active investigation, then the subject is lost to follow-up.

If a patient stops the research prematurely, the end of trial form will be completed for the patient and the reasons for leaving the study recorded.

If some participants stop the research before the end of the study, they will be replaced.

In the event of premature discontinuation of the research, exclusion from the research, withdrawal of consent or abandonment of the research, management will continue in the usual way, as part of their routine care.

8.7. RESEARCH CONSTRAINTS AND POTENTIAL COMPENSATION FOR PARTICIPANTS

Patients included in the study are prohibited from participating in any other interventional research at the same time in order to eliminate any interference. There is no exclusion period at the end of the study.

No compensation will be paid to the patient.

9. MANAGEMENT OF ADVERSE EVENTS / ADVERSE REACTIONS / INCIDENTS

This protocol corresponds to research involving the human person "with minimal risks and constraints" (category 2 according to article L1121 of the CSP) which no longer requires investigators to report "serious" adverse events to the sponsor.
The study procedure (use of a humanoid robot) is risk-free.

Thus, SAEs will not be reportable to the sponsor.

The investigator should report any potential adverse events or incidents to the various **health vigilance networks** applicable to each product or procedure concerned

(pharmacovigilance, medical device vigilance, hemovigilance, standard care vigilance...) in accordance with the regulations in force.

When reporting an adverse event/incident, the investigator should specify that the patient is enrolled in a clinical trial and clearly identify the clinical trial involved.

In the event of a potential adverse event or occurrence of an incident that may significantly affect patient safety and/or the benefit/risk balance and/or the conduct of the H2R2 study, the investigator will immediately notify the sponsor at the contact information listed below:

.

Sponsor: CHU de TOULOUSE

Name of the person in charge of the vigilance : Pascale OLIVIER -ABBAL

Fax : 05 61 77 84 11

E-mail: vigilance.essaiscliniques@chu -toulouse.fr

10. STATISTICAL ASPECTS

10.1. SIZING CALCULATION OF STUDY

If we consider as an acceptability criterion "using the robot for at least 1 hour per week", we hope that this acceptability criterion can be reached by at least half of the participants. It is also considered that if less than 15% of the patients reach this average time of use, the acceptability is clearly insufficient for this version of the robot.

A Fleming A'Hern single-stage design can be applied, where we want to be able to reject the null hypothesis (A'Hern RP. Sample size tables for exact single -stage phase II designs. Stat med 2001;20:859-66):

H0 = {Pr(use robot for at least 1 hr per week) ≤ 15%), with a

possible alternative hypothesis:

H1 = {Pr(use robot for at least 1 h per week) ≥ 50%), with power of

80% and risk α of 5%.

The number of patients needed to be included is 10 patients, and the null hypothesis would be rejected if at least 4 patients out of a total of 10 use the robot for at least 1 hour per week .

In other words, if in a sample of 10 patients, less than 4 patients use the robot for at least 1 hour per week, the acceptability will be considered insufficient to continue the evaluation of the robot in this version towards comparative studies of higher level of evidence.

10.2. STATISTICAL METHODS USED

Missing data will be described and outliers investigated using logical checks. Missing and outlier data will be corrected where possible. The analysis will be done on all participants who agreed to participate in the study.

A flow-chart will be made to describe the inclusions and possible premature exits (with the reasons for exit).

For the primary endpoint, the number of weekly hours of robot use will be described week by week for each patient, which will make it possible to describe the temporal evolution of

acceptability. The quantitative distribution of this indicator will be described in detail (number, mean, standard deviation, minimum, quartiles, median and maximum).
A linear mixed model will be applied to estimate the average temporal evolution of the number of weekly hours of robot solicitation in the entire study population.

The same analysis approach will be applied for the judgment criteria measured repeatedly during the follow - up: user experience, fun and emotions felt.

For the secondary endpoints measured at the end of the study, a simple descriptive analysis of the distribution will be performed (number of patients, mean, standard deviation, minimum, quartiles, median and maximum).

11. RESEARCH MONITORING

However, a scientific committee composed of the investigating team, the sponsor and the methodologist may meet to monitor the progress of the study and discuss any difficulties encountered.

12. ACCESS RIGHTS TO SOURCE DATA AND DOCUMENTS

12.1. ACCESS TO DATA

Acceptance of participation in the protocol implies that the investigators will make available the documents and individual data strictly necessary for the monitoring, quality control and auditing of the research, to persons having access to these documents in accordance with the legislative and regulatory provisions in force.

12.2. SOURCE DATA

All information contained in original documents, or authenticated copies of such documents, relating to clinical examinations, observations or other activities conducted in the course of research and necessary for the reconstruction and evaluation of the research. The documents in which the source data are recorded are called the source documents.

In this research, the source documents will be the patients' medical records, the data collected on the robot and on the tablet. The results of the speech therapy evaluation will be listed in the observation book, so these data will not have any source data.

12.3. CONFIDENTIALITY OF DATA

In accordance with the legislative provisions in force, the persons having direct access to the source data will take all the necessary precautions to ensure the confidentiality of the information relating to the investigational medicinal products, to the research, to the persons who take part in it and in particular as regards their identity as well as the results obtained. These persons, as well as the investigators themselves, are subject to professional secrecy.

During the research or at its conclusion, the data collected on the persons who lend themselves to it and transmitted to the sponsor by the investigators (or any other specialized intervening parties) will be made anonymous. Under no circumstances should the names or addresses of the individuals concerned be made public.

Only the first letter of the subject's first and last name will be recorded, along with an inclusion number.

The sponsor will ensure that each person who participates in the research has given written consent for access to his or her personal data that is strictly necessary for the quality control of the research.

13. QUALITY CONTROL AND ASSURANCE

13.1. INSTRUCTIONS FOR THE COLLECTION OF DATA COLLECTION

All information required by the protocol must be recorded in the observation books and an explanation must be provided for any missing data. Data should be collected as they are obtained, and transcribed into these notebooks in a neat and legible manner.

The data are collected on a paper observation notebook

13.2. QUALITY CONTROL

A clinical research associate mandated by the sponsor regularly visits each investigating center, at the time of the implementation of the research, once or several times during the research according to the rhythm of the inclusions and at the end of the research. During these visits, and in accordance with the risk-based monitoring plan (participant, logistics, impact, resources), the following elements will be reviewed

- informed consent,
- compliance with the research protocol and the procedures defined therein,
- quality of data collected in the observation book: accuracy, missing data, consistency of data with source documents (medical records, appointment books, original laboratory results, etc.),

A written monitoring report will be prepared for each visit.

13.3. DATA MANAGEMENT

The investigator ensures that the anonymity of each person participating in the study is guaranteed. No information allowing the identification of individuals is communicated to third parties other than those authorized by law to hold this information (and who are bound by professional secrecy).

The information is collected for each subject on an observation notebook filled out by the investigators of the center and the speech therapists. The clinical and behavioral data collected will be reported in a paper observation book.

A source document (existing medical record and observation book created for the needs of the study) is kept for each subject participating in the study; the observation and medical follow-up concerning the study will be recorded in this document. This source document will be kept in the ENT department of the Pierre Paul Riquet Hospital.

All study data are kept on computer by the investigating physicians or their delegates.

Each patient's observation book will be kept in the ENT department of the Pierre Paul Riquet Hospital under the control of the principal investigator, and will be reviewed by the on-site monitoring CRA before being sent to the Study Technician for entry.

Data entry will be supervised by the study's methodologist and statistician. Data entry procedures will be defined jointly with the Project Manager.

The data collected on the robot will be stored on an external computer system, respecting the anonymity methods of the study. This data will be kept for a minimum of 15 years.

The statistical analysis will be carried out on the primary endpoint but also on the secondary endpoints after prior verification of the quality and completeness of the data collected.

13.4. AUDIT AND INSPECTION

An audit may be carried out at any time by persons mandated by the sponsor and independent of those conducting the research. The purpose of the audit is to verify the safety of the participants and the respect of their rights, the respect of the applicable regulations and the reliability of the data.

An inspection may also be carried out by a competent authority (ANSM for France or EMA in the context of a European trial, for example).

Both auditing and inspection can be applied to all stages of research, from protocol development to publication of results and classification of data used or generated in the research.

The investigators agree to comply with the sponsor's requirements for an audit and the competent authority for an inspection of theresearch.

14. ETHICAL AND REGULATORY CONSIDERATIONS

The sponsor and the investigator(s) undertake that this research will be carried out in accordance with the law n°2012-300 of March 5, 2012 relating to research involving the human person, as well as in accordance with the Good Clinical Practices (I.C.H. version 4 of November 9, 2016 and decision of November 24, 2006) and the declaration of Helsinki (which can be found in its integral version on the site http://www.wma.net).

The research is conducted in accordance with this protocol. Except in emergency situations requiring the implementation of specific therapeutic procedures, the investigator(s) undertake to respect the protocol in all respects, in particular with regard to the collection of consent and the notification and follow-up of serious adverse events.

This research has received the favorable opinion of the Comité de Protection des Personnes (CPP) of Ile de France 1.

The Toulouse University Hospital, promoter of this research, has taken out a civil liability insurance policy with Lloyds Insurance in accordance with the provisions of the Public Health Code.

The data recorded on the occasion of this research are subject to computerized processing at CHU de Toulouse in compliance with the law n°78 -17 of January 6, 1978 modified by law n°2018 -493 of June 20, 2018 relating to data processing, files and freedoms as well as the General Regulation on Data Protection, regulation n° 2016/679 adopted by the European Parliament on April 16, 2016

This research falls within the framework of the "Reference Methodology" (MR-001) in application of the provisions of Article 54 paragraph 5 of the amended Act of January 6, 1978 on information, files and freedoms. This change was approved by decision of January 5, 2006, updated on July 21, 2016. The Toulouse University Hospital has signed a commitment to comply with this "Reference Methodology".

- This search is recorded on the website http://clinicaltrials.gov/

CHANGES TO THE PROTOCOL

Any substantial modification, i.e. any modification likely to have a significant impact on the protection of individuals, on the conditions of validity and on the results of the research, on the quality and safety of the products tested, on the interpretation of the scientific documents that support the conduct of the research or on the methods of conducting the research, is the subject of a written amendment that is submitted to the sponsor; the sponsor must obtain a favorable opinion from the CPP and, where applicable, authorization from the ANSM prior to its implementation.

Non-substantial changes, i.e., those that do not have a significant impact on any aspect of the research, are communicated to the PPC for information purposes.

All modifications are validated by the sponsor, and by all the research stakeholders concerned by the modification, before submission to the CPP and, if necessary, to the ANSM. This validation may require the meeting of any committee set up for the research.

All changes to the protocol must be communicated to all investigators participating in the research. Investigators agree to abide by the contents of the protocol.

Any change that modifies the management of the participants or the benefits, risks and constraints of the research is the subject of a new information note and a new consent form, the collection of which follows the same procedure as that mentioned above.

15. PRESERVATION OF RESEARCH DOCUMENTS AND DATA

The following documents related to this research are archived by the investigator in accordance with Good Clinical Practice:

- *for a period of 1 5 years following the end of the research* :
  - The protocol and any amendments to the protocol
  - Observation books (copies)
  - Source records of participants who have signed a consent
  - All other documents and correspondence related to the research
  - Original signed informed consents from participants

All these documents are the responsibility of the investigator during the regulatory archiving period. No removal or destruction can be made without the sponsor's agreement. At the end of the regulatory archiving period, the sponsor will be consulted for destruction. All data, documents and reports may be subject to audit or inspection.

16. FINAL REPORT

Within one year of the end of the research or its interruption, a final report will be drawn up and signed by the sponsor and the investigator. This report will be made available to the competent authority. The sponsor will transmit the results of the research to the CPP and, if applicable, to the ANSM in the form of a summary of the final report within one year of the end of the research.

17. RULES FOR PUBLICATION

17.1. COMMUNICATIONS SCIENTIFICS

The analysis of the data provided by the investigating centers is carried out by the Toulouse University Hospital. This analysis gives rise to a written report which is submitted to the promoter, who will transmit it to the Comité de Protection des Personnes and to the competent authority.

Any written or oral communication of the results of the research must have the prior approval of the coordinating investigator and, if applicable, any committee established for the research.

The coordinating/principal investigator agrees to make available to the public the results of the research, both negative and inconclusive as well as positive.

The publication of the main results mentions the name of the sponsor, of all the investigators who included or followed participants in the research, of the methodologists, biostatisticians and data managers who participated in the research, of the vigilants who participated in the analysis of the safety of the participants, of the members of the committee(s) set up for the research and the source of funding. The international rules for writing and publishing will be taken into account (*The Uniform Requirements for Manuscripts* of the ICMJE, April 2010).

17.2. COMMUNICATION OF RESULTS TO PARTICIPANTS

In accordance with the law n°2002 -303 of March 4, 2002, the participants are informed, at their request, of the global results of the research.

17.3. TRANSFER OF DATA

The management of the data is ensured by the Toulouse University Hospital. The conditions of transfer of all or part of the research database are decided by the research sponsor and are the subject of a written contract.

BIBLIOGRAPHIC REFERENCES

1. Chella A, Pipitone A. A cognitive architecture for inner speech. Cognitive Systems Research. 2020 Jan;59:287-92.
2. Di Nuovo A, Varrasi S, Lucas A, Conti D, McNamara J, Soranzo A. Assessment of Cognitive skills via Human-robot Interaction and Cloud Computi ng. Journal of Bionic Engineering. Springer Singapore; 2019 May;16(3):526-39.
3. Tanioka R, Locsin R, Yasuhara Y, Tanioka T. Potential Legal Issues and Care Implications during Care - Prevention Gymnastic Exercises for the Elderly Using Pepper in Long Term Health Care Facilities. Intelligent Control and Automation. Scientific Research Publishing; 2018 Aug 24;09(03):85 -93.
4. Aaltonen I, Arvola A, Heikkilä P, Lammi H. Hello Pepper, May I Tickle You? New York, New York, USA: ACM Press; 2017. pp. 53-4.
5. Schicchi D, Pilato G. A Social Humanoid Robot as a Playfellow for Vocabulary Enhancement. IEEE; pp. 205-8.
6. Schicchi D, International GP2SI, 2018. A Social Humanoid Robot as a Playfellow for Vocabulary Enhancement - IEEE Conference Publication. ieeexplorei eeeorg
7. Piezzo C, Suzuki K. Design of an accompanying humanoid as a walking trainer for the elderly. IEEE; pp. 467-72.
8. Tanaka F, Isshiki K, Takahashi F, Uekusa M, Sei R, Hayashi K. Pepper learns together with children: Development of an educational application. IEEE; pp. 270 -5.
9. Pandey AK, Gelin R, Automation ARIR, 2018. Pepper: The First Machine of Its Kind. Researchgatenet
10. Diehl J, Schmitt L, Villano M, Crowell C. The clinical use of robots for individuals with autism spectrum disorders: a critical review. Research in Autism Spectrum Disorders; 6 (2012) 249-26211. Ismail L, Shamsudina S, Yussofa H, Hanapiahc F, Zaharid N. Robot-based Intervention Program for Autistic Children with Humanoid Robot NAO: Initial Response in Stereotyped Beha vior; Procedia Engineering 41 (2012) 1441 - 1447

Bibliographic references for standardized questionnaires

[1] Agarwal, R., & Karahanna, E. (2000). Time flies when youare having fun: Cognitive absorption and beliefs about IT usage. MIS Quarterly, 24(4), 665 -694. doi:10.2307/3250951

-[7] Desmet, P.M.A., Vastenburg, M.H., Romero Herrera, N. (2016). Mood measurement with Pick - A)Mood: review of current methods and design in a pictorial self -report scale. J. of Design Research, 14(3), 241-279

[9] Heerink, M., Krose, B., Evers, V., & Wielinga, B. (2010). Assessing acceptance of assistive social agent technology by older adults: The Almere Model. International Journal of Social Robotics, 2(4), 361 -375.

[12] Lallemand, C., Gronier, G., (2018). UX design methods: 30 fundamental methods for designing optimal experiences. Eyrolles.

[13] Lallemand, C. & Koenig, V. (2017). "How Could an Intranet be Like a Friend to Me?" - Why Standardized UX Scales Donat Always Fit. Proceedings of ECCE 2017, Umea, Sweden.

[14] Lallemand, C., Koenig, V., Gronier, G., & Mar5n, R. (2015). Creation and validation of a French version of the AGrakDif questionnai re for user experience assessment of interactive systems, European Journal of Applied Psychology. doi:10.1016/j.erap.2015.08.002 [16] Moon, J. W., & Kim, J. G. (2000). Extending the TAM for a world-wide-web context. Information & Management, 38, 217-230.

[17] Read, J., MacFarlane, S., Casey, C., (2002) Endurability, Engagement and Expectations: Measuring Childrenas Fun

[15] Sánchez-Morales, A., Durand-Rivera, J. A., & Martínez -González, C. L. (2020). Usability evaluation of atangible user interface and serious game for identification of cognitive deficiencies in preschool children. *International Journal of Advanced Computer Science and Applications* , *11*(6), 486- 493.

[16] Xu, D., Read, J. C., Sim, G., & McManus, B. (2009, June). Experience it, draw it, rate it: capturing children's experiences with their drawings. In Proceedings of the 8th International Conference on Interaction Design and Children (pp. 266-270).
